# Combining CO_2_ reduction with propane oxidative dehydrogenation over bimetallic catalysts

**DOI:** 10.1038/s41467-018-03793-w

**Published:** 2018-04-11

**Authors:** Elaine Gomez, Shyam Kattel, Binhang Yan, Siyu Yao, Ping Liu, Jingguang G. Chen

**Affiliations:** 1 Department of Chemical Engineering, Columbia University, New York, NY 10027 USA; 2 Chemistry Department, Brookhaven National Laboratory, Upton, NY 11973 USA

## Abstract

The inherent variability and insufficiencies in the co-production of propylene from steam crackers has raised concerns regarding the global propylene production gap and has directed industry to develop more on-purpose propylene technologies. The oxidative dehydrogenation of propane by CO_2_ (CO_2_-ODHP) can potentially fill this gap while consuming a greenhouse gas. Non-precious FeNi and precious NiPt catalysts supported on CeO_2_ have been identified as promising catalysts for CO_2_-ODHP and dry reforming, respectively, in flow reactor studies conducted at 823 K. In-situ X-ray absorption spectroscopy measurements revealed the oxidation states of metals under reaction conditions and density functional theory calculations were utilized to identify the most favorable reaction pathways over the two types of catalysts.

## Abstract (web summary)

The oxidative dehydrogenation of propane by CO_2_ (CO_2_-ODHP) can potentially fill the gap of propylene production while consuming a greenhouse gas. Here, the authors identify non-precious FeNi and precious NiPt catalysts supported on CeO_2_ as promising catalysts for CO_2_-ODHP and dry reforming, respectively, in flow reactor studies.

## Introduction

**Fig. 1:**
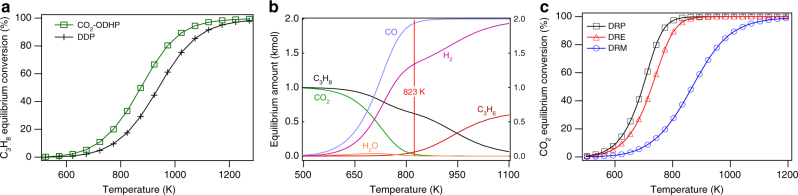
Thermodynamic equilibrium plots. Equilibrium calculations were performed through HSC Chemistry 8 software, which utilizes a Gibbs free energy minimization algorithm. **a** C_3_H_8_ equilibrium conversion for CO_2_-ODHP and direct dehydrogenation of propane; **b** product amounts for CO_2_+C_3_H_8_ system and **c** conversions of propane, ethane, and methane dry reforming; all vs. temperature at 1 atm

Propylene is one of the most diverse petrochemical building blocks used for the production of many chemicals (e.g., polypropylene, propylene oxide, and acrylonitrile). The co-production of propylene from steam and fluidized crackers is anticipated to be insufficient to satisfy the rapidly growing demand^1^. Consequently, there is a need for the development of economic on-purpose production techniques to produce additional propylene. The direct dehydrogenation of propane (DDP) is thermodynamically limited and is highly endothermic (Δ*H°*_*r*_ = 29.70 kcal/mol), requiring temperatures that may exceed 973 K for significant propylene yields^2^. In principle, the introduction of CO_2_ as a mild oxidant into the feed alters the dehydrogenation pathway by oxidizing the abstracted hydrogen from the alkane and consequently releasing the heat of reaction that reduces operating temperatures^2,3^. The presence of CO_2_ can also increase the equilibrium conversion of propane by consuming H_2_ through the reverse water gas shift reaction (RWGS), as seen in the thermodynamic calculations in Fig. 1a. Additionally, unlike regular oxidative dehydrogenation with molecular oxygen, CO_2_ as a mild oxidant suppresses over-oxidation and thus minimizes the production of carbon oxides. The reactions of propane and CO_2_ also have the potential to employ two underutilized^4–6^ reactants to supply propylene as well as to mitigate detrimental CO_2_ emissions^7,8^.

The reactions of CO_2_ with propane may occur through two distinct pathways, oxidative dehydrogenation (CO_2_ + C_3_H_8_ → C_3_H_6_ + CO + H_2_O) and dry reforming (3CO_2_ + C_3_H_8_ → 6CO + 4H_2_). The two reactions should occur simultaneously at temperatures around 823 K and above with considerable conversions (Fig. 1b), allowing the formation of both dehydrogenation products (propylene) and reforming products (synthesis gas). The oxidative dehydrogenation of propane by CO_2_ (CO_2_-ODHP) can reach an equilibrium conversion of 33% as opposed to 17% for DDP at 823 K. At that same reaction temperature, as seen in Fig. 1c, CO_2_ equilibrium conversion for the dry reforming of propane (DRP) can reach up to 98% at a temperature 150 K less than that of methane dry reforming (DRM). This in turn would reduce catalyst deactivation due to coking and phase transformations triggered by the relatively high temperatures commonly used in DRM^9,10^. Furthermore, in the CO_2_+C_3_H_8_ system unreacted CO_2_ can remove surface carbon via the Boudouard reaction (CO_2_ + C_s_ → 2CO) at temperatures as low as 773 K with moderate rates^11,12^. Thus, it is of great interest to identify catalysts that can either selectively break the C–H bond to produce propylene or the C–C bonds to generate synthesis gas (CO + H_2_).

Previous work in CO_2_-ODH primarily focuses on supported chromium catalysts^13–15^ as a result of their ability to exist in multiple oxidation states^16^, but implementation is limited due to short lifecycles and high toxicity of chromium^17^. Ni is mainly used for dry reforming, but catalyst deactivation due to severe coking is still a problem^18–20^. To alleviate coke formation, precious metal catalysts (e.g., Rh, Re, Ru) have also been investigated on high surface area Al_2_O_3_^21,22^. However, large scale catalytic conversion of CO_2_ into valuable products would require the development of cost effective, selective, and coking-resistant catalytic systems. While there are studies that examine the CO_2_-ODHP or DRP separately, a thorough examination utilizing supported bimetallic catalysts at a temperature range that allows both pathways to occur is still lacking. Ceria (CeO_2_) is a good choice of oxide support because it has the ability to store/release oxygen and thus may induce direct C–O bond scission of CO_2_, while also providing available lattice oxygen for coke suppression^9,23–25^.

The present work will explore ceria supported bimetallic catalysts, non-precious metal Fe_3_Ni as well as precious metal- based Fe_3_Pt and Ni_3_Pt, that are active at 823 K. In summary, steady-state flow reactor studies indicate that Fe_3_Ni shows promising selectivity toward propylene via the CO_2_-ODHP pathway, whereas Ni_3_Pt is active for the DRP with high selectivity toward CO. Density functional theory calculations of the energetics for the C–H and C–C bond scissions over the two catalysts are in agreement with experimental results.

## Results

### Catalytic evaluation with kinetics and deactivation patterns

Flow reactor studies measuring both CO_2_-ODHP and DRP activity simultaneously are summarized in Table 1 along with CO chemisorption values. All catalysts were synthesized via incipient wetness impregnation of metals onto commercially obtained CeO_2_ (35–45 m^2^/g, Sigma Aldrich). For additional details see Methods section or Supplementary Methods section. Results for conversions and product selectivity following time on stream for all catalysts are shown in Supplementary Fig. 1. The monometallic Ni_1_ catalyst exhibits 12%–87% C_3_H_6_ and reforming selectivity, respectively, with minimal cracking products (CH_4_ and C_2_ hydrocarbons), while the Fe_3_ monometallic catalyst is not active for either reaction. The bimetallic system, Fe_3_Ni, however, at steady-state demonstrates propylene production from the CO_2_-ODHP reaction, corresponding to 58.2% C_3_H_6_ selectivity. The differences among the propylene yields on a C_3_H_8_ basis provided in Supplementary Table 1 of Fe_3_Ni (1.6% C_3_H_6_ yield) and the respective monometallics (C_3_H_6_ yield of 0.4% over Ni and 0.2% over Fe) indicate that there is a synergistic effect from the formation of the bimetallic Fe_3_Ni catalyst.

**Table 1:** Catalyst flow reactor results for CO_2_ + C_3_H_8_ reaction

	Fe_3_Ni	Fe_3_Pt	Ni_3_Pt	*Ni_3_Pt	Ni_1_	Ni_3_	Pt_1_
CO uptake (μmol g^−1^)	31.9	31.5	50.1	–	13.1	37.7	16
Conversion (%)							
CO_2_	4	2.6	39.4	7.8	9.3	32.8	4.2
C_3_H_8_	2.7	1.1	11.6	2.2	3	9.6	1.6
TOF (site^−1^ min^−1^)							
CO_2_	5.7	3.5	37.5	–	31.9	40.2	8.1
C_3_H_8_	3.4	1.5	10.5	–	8.9	11.4	2.8
Selectivity (%)							
CO	40.2	65.1	96.2	87.8	86.8	94.9	77
C_3_H_6_	58.2	32	2.8	11	12.3	2.9	21.2
CH_4_	0.8	1.3	0.83	0.9	0.6	2.11	0.8
C_2_H_6_	0	0	0.1	0	0.24	0.05	0.9
C_2_H_4_	0.8	1.6	0	0.3	0	0.06	0
Yield (%)							
CO	1.1	0.7	11.1	2	2.6	9.1	1.3
C_3_H_6_	1.6	0.3	0.3	0.2	0.4	0.3	0.4

10 mL/min each reactant at 823 K with Ar diluent (20 mL/min) and 100 mg of catalyst (16–20 mesh). Catalysts marked with an asterisk indicate that the sample was diluted to achieve comparable C_3_H_8_ reactant conversion to Fe_3_Ni. Values are obtained by averaging data from 10–12 h. Selectivity and yield are on a C_3_H_8_ basis (including only carbonaceous species). Catalysts are synthesized by atomic ratios corresponding to a 1.67 wt.% Pt_1_ basis, thus the weight percent of Fe_3_, Ni_1_, and Ni_3_ are 1.43, 0.5, and 1.5, respectively. The nomenclature assigned by subscripts such as in Fe_3_Ni means that there are three atoms of Fe for every atom of Ni

Exchanging Ni in the Fe_3_Ni catalyst with precious metal Pt (Fe_3_Pt) roughly reduces the activity by half, decreases the selectivity toward C_3_H_6_ to 32%, and is unstable compared to Fe_3_Ni (Supplementary Fig. 2). The other precious metal bimetallic catalyst, Ni_3_Pt, primarily performs the DRP reaction with 39% CO_2_ conversion, a robust selectivity toward CO of 88% at comparable reactant conversions (Supplementary Table 2) and is more stable compared to monometallic Ni_3_ (Supplementary Fig. 3). Thus, when Ni is coupled with non-precious Fe at a ratio of 1:3, higher dehydrogenation activity can be achieved and propylene is produced. In contrast, when Ni is alloyed with precious metal Pt, reforming activity is enhanced compared to monometallic Ni_3_. Further analysis, such as the comparison of CeO_2_ supported Ni_3_Pt with Ni_3_Fe and Fe_3_Ni catalysts along with CO selectivity following CO_2_ conversion plots can be found in Supplementary Notes 1 and 2, respectively.

**Fig. 2:**
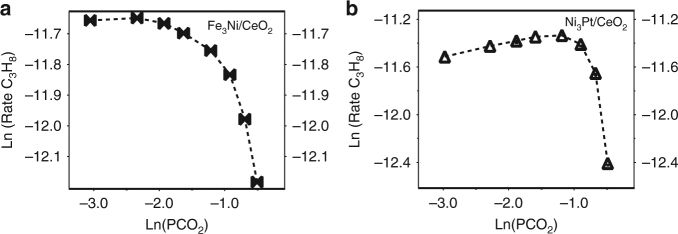
Effect of CO_2_ partial pressure on the propane production rate. Plots for **a** Fe_3_Ni and **b** Ni_3_Pt. Total system pressure is 1 atm

Kinetic studies examining the influence of the reactant partial pressure and the reaction temperature on the activity of Fe_3_Ni and Ni_3_Pt were conducted to further evaluate the differences between the two types of catalysts. The apparent activation energies were derived by measuring production rates in the temperature range of 803–843 K. Over Fe_3_Ni, the activation barrier for propane CO_2_ oxidative dehydrogenation was found to be 115 kJ mol^−1^, while the activation barrier for reforming over Ni_3_Pt was 119 kJ mol^−1^. Arrhenius-type plots and additional values are available in Supplementary Fig. 4 and Supplementary Table 3, respectively. As seen in Fig. 2a, the reactant consumption rate of C_3_H_8_ for the Fe_3_Ni CO_2_-ODHP catalyst was initially unaffected by increasing the partial pressure of CO_2_ but upon reaching a C_3_H_8_:CO_2_ ratio of 1:1, the rate started to decline. The reforming catalyst, on the other hand, was positively influenced by the partial pressure of CO_2_ until the aforementioned ratio of 1:6. Increasing the C_3_H_8_ partial pressure produced similar trends and are shown in Supplementary Fig. 5. The declining rates signify that there are less catalytic sites available for one reactant when the other is in excess, indicative of competitive adsorption of adsorbates and/or surface intermediates. Particularly, the rates for both reactants decrease at high propane partial pressure, suggesting that as the reaction progresses intermediates from propane block surface sites and lead to a loss in activity.

To further evaluate how different reaction pathways may influence deactivation patterns, both thermogravimetric (TGA) and energy dispersive spectroscopy (EDS) experiments were conducted and results are provided in Supplementary Figs 6 and 7, respectively. The TGA results indicate that the Fe_3_Ni catalyst only loses less than half a percent of its original mass, therefore, it is unlikely that the main deactivation pathway is due to coking. The EDS of the spent Fe_3_Ni sample shows small regions of higher Ni content, and to a lesser extent regions with higher Fe. However, in-situ XRD measurements do not reveal obvious agglomeration formation during reaction, and the absence of metal diffraction peaks suggests that the metal particles are most likely less than 2 nm in size (Supplementary Fig. 8). The Ni_3_Pt catalyst loses about 8% of its original mass but does not illustrate signs of sintering. However, the coking over the Ni_3_Pt catalyst at comparable propane conversion to Fe_3_Ni is not significant.

### Oxidation states by in situ XANES

**Fig. 3:**
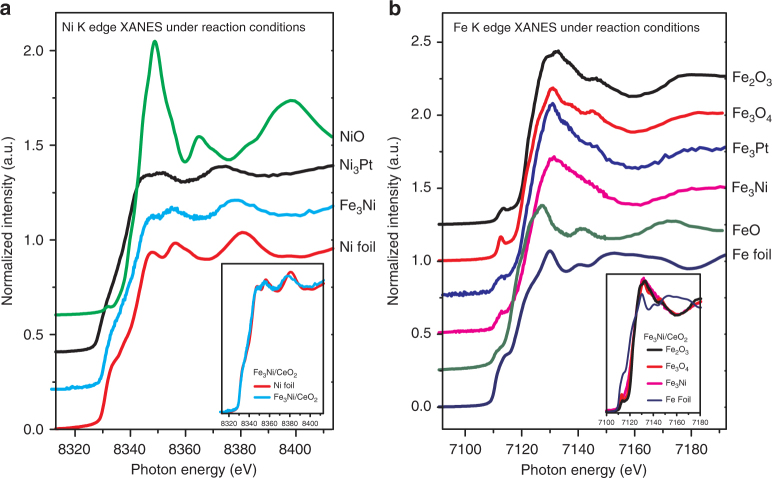
In-situ XANES spectra. **a** Ni and **b** Fe K edges of all the bimetallic catalysts with respective references. The insets show more detailed comparison of Fe_3_Ni with model compounds

In situ X-ray absorption near edge spectroscopy (XANES) measurements were conducted in order to identify the local environment of the metals under reaction conditions, as shown in Fig. 3. Additional details are available in Supplementary Note 3. The XANES data identified that under reaction conditions the Ni_3_Pt catalyst consisted of metallic Pt (Supplementary Fig. 9) and that both the Fe_3_Ni and Ni_3_Pt catalysts consisted of metallic Ni (Fig. 3a). On the other hand, the Fe in the Fe_3_Ni catalyst was in the oxidized form. The extended X-ray absorption fine structure (EXAFS) fitting of Fe_3_Ni suggested the presence of an inserted oxygen through Fe–O–Fe as well as Fe–O bonds (Supplementary Table 4). Theofanidis et al. and Kim et al. studied DRM over higher loading Ni–Fe catalysts (8 wt.% Ni-5 wt.% Fe, and 8.8 wt.% Ni-2.1 wt.% Fe, respectively) supported on magnesium aluminate and they also observed oxidized Fe under in situ conditions but in an oxidation state of 2+^26,27^. For the Ni_3_Pt catalyst, the EXAFS fitting indicates that the coordination number of the Pt–Pt and Pt–Ni bonds is 3.4 and 6.4, respectively, confirming the formation of the Pt–Ni bimetallic bond.

### Reaction pathways and DFT calculations

**Fig. 4:**
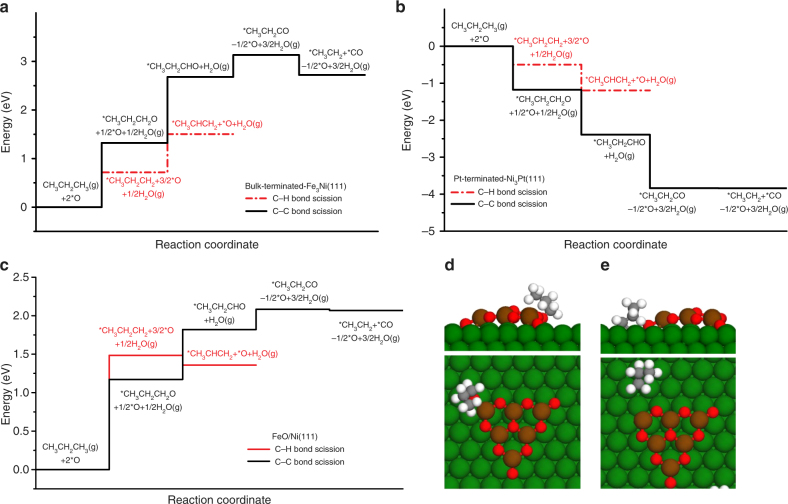
DFT calculated energy profiles for the oxidative C–H and C–C bond scission pathways. **a** Bulk Fe_3_Ni(111) surface, **b** Pt-terminated Ni_3_Pt(111) surface, and **c** FeO/Ni(111) interface as well as the optimized geometries of **d** CH_3_CH_2_CH_2_O and **e** CH_3_CH_2_CH_2_ on FeO/Ni(111)

Density functional theory (DFT) calculations were performed on bulk-terminated-Fe_3_Ni(111) and Pt-terminated-Ni_3_Pt(111) surfaces (Supplementary Fig. 10) to further gain insight into the potential reaction pathways for the oxidative C–H and C–C bond cleavage of propane to form *CH_3_CHCH_2_+H_2_O(g) and *CH_3_CH_2_+*CO+H_2_O(g), respectively. In these calculations, the surfaces are first modified by *O atoms assuming that *CO_2_ dissociates to form *CO + *O. The DFT optimized geometries in Supplementary Fig. 11 show that the intermediates *CH_3_CH_2_CH_2_O, *CH_3_CH_2_CHO, and *H_2_O interact with the surfaces via the oxygen atoms while other intermediates *CH_3_CH_2_CH_2_, *CH_3_CHCH_2_, *CH_3_CH_2_, and *CO interact with the surfaces via the carbon atoms. It is noted that, even though the binding configurations of intermediates are similar on both surfaces, all the intermediates bind more strongly on bulk-terminated-Fe_3_Ni(111) than on Pt-terminated-Ni_3_Pt(111) (Supplementary Tables 5 and 6). The DFT calculated binding energies were then used to calculate the change in energy for the oxidative C–H and C–C bond scission of propane. On bulk-terminated-Fe_3_Ni(111), Fig. 4a shows that the pathway for the oxidative C–H bond cleavage lies lower in energy than that for the C–C bond scission. In contrast, as shown in Fig. 4b on Pt-terminated-Ni_3_Pt(111), the pathway for the C–C bond cleavage lies lower in energy than that for the C–H bond.

Overall the DFT results reveal that the C–C bond cleavage pathway is preferred on Ni_3_Pt(111), while bulk-terminated-Fe_3_Ni(111) favors the C–H bond cleavage pathway. Kinetically, this is also the case based on the comparison of activation energies (Supplementary Table 7). According to the DFT calculations, on Pt-terminated-Ni_3_Pt(111), the *O insertion reaction (*CH_3_CH_2_CH_2_ + *O → *CH_3_CH_2_CH_2_O + *) along the C–C bond cleavage pathway (*∆E* = −0.75 eV and *E*_a_ = 1.07 eV) is thermodynamically and kinetically more favorable than the oxidative dehydrogenation reaction (*CH_3_CH_2_CH_2_ + *O → *CH_3_CHCH_2_ + *OH) along the C–H bond cleavage pathway (*∆E* = −0.51 eV and *E*_a_ = 1.33 eV). In contrast, on bulk-terminated-Fe_3_Ni(111), the oxidative dehydrogenation reaction (*∆E* = 0.29 eV and *E*_a_ = 1.02 eV) is more favorable than the *O insertion reaction (*∆E* = 0.43 eV and *E*_a_ = 3.30 eV). These DFT predictions are in agreement with experimental observations, suggesting that the bulk-terminated-Fe_3_Ni(111) surface promotes the oxidative C–H bond cleavage of propane to form *CH_3_CHCH_2_ while the Pt-terminated-Ni_3_Pt(111) surface promotes the C-C bond cleavage of propane to form *CO.

To account for the potential FeO–Ni interfacial active sites based on the in situ experimental observation of oxidized Fe in the Fe_3_Ni catalyst, further DFT calculations were carried out to investigate the pathways for the oxidative C–H and C–C bond cleavage of propane on the FeO/Ni(111) interface. For the FeO_*x*_ clusters supported on Ni(111), both Fe_6_O_9_ and Fe_3_O_3_ clusters on three layer 7 × 7 Ni(111) and 5 × 5 Ni(111) surfaces (Supplementary Fig. 12) were considered. The oxygenated species (*O, *CO, *CH_3_CH_2_CH_2_O, *CH_3_CH_2_CHO, and *CH_3_CH_2_CO) prefer to adsorb at the interfacial sites while *C_*x*_H_*y*_ species (*CH_3_CH_2_CH_2_, *CH_3_CHCH_2_, and *CH_3_CH_2_) most favorably adsorb on Ni(111) sites (Supplementary Table 8 and Supplementary Fig. 13). The energy diagram in Fig. 4c, calculated based on the DFT obtained binding energies of the potential intermediates, show that the first steps in oxidative C–C and C–H bond cleavage pathways are competitive. The subsequent step to form *CH_3_CHCH_2_ is downhill in energy along the oxidative C–H bond cleavage pathway; in contrast, the subsequent steps are uphill in energy along the oxidative C–C bond cleavage pathway. Again, such thermodynamic predictions are fully supported by the calculated *E*_a_, showing that the oxidative dehydrogenation reaction (*∆E* = −0.40 eV and *E*_a_ = 0.29 eV) is highly favorable over the *O insertion reaction (*∆E* = 0.01 eV and *E*_a_ = 2.13 eV) on the Fe_3_O_3_/Ni(111) surface. This indicates that the oxidative dehydrogenation pathway should be more favorable than reforming, consistent with experimental observation.

Finally, on the three surfaces studied the desorption of *CO is expected to be a facile process due to the contribution of entropy at 823 K. *C_2_H_5_ is one of the reaction intermediates that undergoes O-insertion, C–H and C–C bond scission reactions to eventually produce CO and H_2_. The *O species on Pt-terminated-Ni_3_Pt(111) react with *C_*x*_H_*y*_ to form the *C_*x*_H_*y*_O intermediate, which promotes the C–C bond scission. In contrast, the more stable *O on bulk-terminated-Fe_3_Ni(111) and the FeO/Ni(111) interface are expected to remain on the surface, which facilitates the selective C–H bond scission of propane to produce propylene.

## Discussion

Overall, the oxidative dehydrogenation of propane with CO_2_ has the potential to combine two underutilized^4–6^ reactants to produce propylene or syngas. Two types of bimetallic catalysts have been identified for the CO_2_ + C_3_H_8_ system. The DFT calculation results indicate that the bulk Fe_3_Ni(111) surface and the FeO/Ni(111) interface should favor C–H bond scission for the CO_2_-ODHP pathway, whereas the Pt-terminated Ni_3_Pt(111) surface should favor the C–C bond cleavage for the DRP pathway. Flow reactor results are consistent with the DFT calculations as it was observed that the Fe_3_Ni catalyst is selective for propylene production, while the Ni_3_Pt catalyst shows good activity and CO selectivity. The oxidation states of the different metals provided by in situ XANES measurements reveal that Fe_3_Ni consists of oxidized Fe and metallic Ni. Future efforts should be geared toward enhancing propylene yield through the discovery of more stable and selective catalytic materials.

## Methods

### Density functional theory calculations

Spin polarized^28,29^ density functional theory (DFT) calculations were performed as an attempt to elucidate the possible pathways of C–C and C–H bond cleavage of propane over Fe_3_Ni(111), Ni_3_Pt(111) surfaces, and FeO/Ni(111) interface using the Vienna Ab Initio Simulation Package (VASP) code^30,31^. Projector augmented wave potentials were used to describe the core electrons with the generalized gradient approximation (GGA)^32,33^ using PW91 functionals^34^. The Kohn−Sham one-electron wave functions were expanded by using a plane wave basis set with a kinetic energy cutoff of 400 eV. The Brillouin zone was sampled using a 3 × 3 × 1 k-point grid in the Monkhorst−Pack scheme^35^. Ionic positions were optimized until Hellman–Feynman force on each ion was smaller than 0.02 eV/Å. The transition state of a chemical reaction was located using the climbing image nudged elastic band (CI-NEB) method implemented in VASP^36^. The activation energy (*E*_a_) of a chemical reaction is defined as the energy difference between the initial and transition states while the reaction energy (Δ*E*) is defined as the energy difference between the initial and final states.

### Catalyst preparation and flow reactor studies

The catalysts evaluated in this study were synthesized through incipient wetness impregnation of metals onto commercially obtained CeO_2_ (35–45 m^2^/g, Sigma-Aldrich). Flow reactor experiments were performed under atmospheric pressure utilizing a 1/4” quartz U-shaped reactor. All catalysts were reduced at 723 K for 1 h under a 1:1 H_2_/Ar flow (40 mL/min total). Subsequently, the temperature was increased and held at 823 K in the presence of 1:1:2 CO_2_, C_3_H_8_, and Ar for 12 h. Apparent activation barrier and reaction order experiments were conducted at slightly different reaction conditions to ensure operation in a true intrinsic kinetic regime and minimize transport effects. XANES measurements were conducted using a custom in situ micro-channel cell holding ~200 mg of catalyst (60–80 mesh) and a 4-channel vortex fluorescence detector.

### Data availability

The data that support the findings of this study are available from the corresponding author upon request.

## Electronic supplementary material

(DOCX 8938 kb)

Peer Review File(PDF 2247 kb)

## References

[1] Plotkin, J. S. The propylene gap: how can it be filled? http://www.acs.org/content/acs//en/pressroom/cutting-edge-chemistry/the-propylene-gap-how-can-it-be-filled.html (2015).

[2] Wang S, Zhu ZH (2004). Catalytic conversion of alkanes to olefins by carbon dioxide oxidative dehydrogenation—a review. Energy Fuels.

[3] Ansari MB, Park SE (2012). Carbon dioxide utilization as a soft oxidant and promoter in catalysis. Energy Environ. Sci..

[4] Centi, G., Perathoner, S. & Iaquaniello, G. in *CO*_*2*_*: A Valuable Source of Carbon* (eds De Falco, M., Iaquaniello, G & Centi, G.) Ch. 2 (Springer-Verlag, London, 2013).

[5] Sloan, M. & Wilczewski, W. Propane market outlook. http://www.afdc.energy.gov/uploads/publication/2016_propane_market_outlook_1_.pdf (2013).

[6] Sloan, M. Propane market outlook. http://www.afdc.energy.gov/uploads/publication/2013_propane_market_outlook.pdf (2016).

[7] Goddard, P. B., Yin, J., Griffies, S. M. & Zhang, S. An extreme event of sea-level rise along the Northeast coast of North America in 2009–2010. *Nat. Commun*. **6**, 6346 (2015). 10.1038/ncomms7346 25710720

[8] Pachauri, R. K. et al. (eds) *Climate Change: Synthesis Report.**Contribution of Working Groups I, II and III to the Fifth Assessment Report of the Intergovernmental Panel on Climate Change* (IPCC, 2014).

[9] Pakhare D, Spivey J (2014). A review of dry (CO_2_) reforming of methane over noble metal catalysts. Chem. Soc. Rev..

[10] Tang P, Zhu Q, Wu Z, Ma D (2014). Methane activation: the past and future. Energy Environ. Sci..

[11] Osaki T, Mori T (2006). Kinetics of the reverse-Boudouard reaction over supported nickel catalysts. React. Kinet. Catal. Lett..

[12] Lim JY, McGregor J, Sederman AJ, Dennis JS (2016). The role of the Boudouard and water-gas shift reactions in the methanation of CO or CO_2_ over Ni/g–Al_2_O_3_ catalyst. Chem. Eng. Sci..

[13] Baek J, Yun HJ, Yun D, Choi Y, Yi J (2012). Preparation of highly dispersed chromium oxide catalysts supported on mesoporous silica for the oxidative dehydrogenation of propane using CO_2_: insight into the nature of catalytically active chromium sites. ACS Catal..

[14] Talati A, Haghighi M, Rahmani F (2016). Oxidative dehydrogenation of ethane to ethylene by carbon dioxide over Cr/TiO_2_–ZrO_2_ nanocatalyst: effect of active phase and support composition on catalytic properties and performance. Adv. Powder Technol..

[15] Takahara I, Chang WC, Mimura N, Saito M (1998). Promoting effects of CO_2_ on dehydrogenation of propane over a SiO_2_ -supported Cr_2_O_3_ catalyst. Catal. Today.

[16] Asghari E, Haghighi M, Rahmani F (2016). CO_2_ oxidative dehydrogenation of ethane to ethylene over Cr/MCM-41 nanocatalyst synthesized via hydrothermal/impregnation methods: influence of chromium content on catalytic properties and performance. J. Mol. Catal. A Chem..

[17] Farrauto, R. J. & Bartholomew, C. H. *Fundamentals of Industrial Catalytic Processes* 2nd edn (Wiley-AIChE, Hoboken, NJ, 2005).

[18] Raberg L (2007). Propane dry reforming to synthesis gas over Ni-based catalysts: influence of support and operating parameters on catalyst activity and stability. J. Catal..

[19] Siahvashi A, Adesina AA (2013). Kinetic study of propane CO_2_ reforming over bimetallic Mo−Ni/Al_2_O_3_ catalyst. Ind. Eng. Chem. Res..

[20] Olafsen A (2006). Light alkanes CO_2_ reforming to synthesis gas over Ni based catalysts. Catal. Today.

[21] Solymosi F, Tolmacsov P, Kedves K (2003). CO_2_ reforming of propane over supported Rh. J. Catal..

[22] Solymosi F, Tolmacsov P, Zakar TS (2005). Dry reforming of propane over supported Re catalyst. J. Catal..

[23] Centi G, Perathoner S (2009). Opportunities and prospects in the chemical recycling of carbon dioxide to fuels. Catal. Today.

[24] Valenzuela RX, Bueno G, Cortés Corberan V, Xu Y, Chen C (2000). Selective oxidehydrogenation of ethane with CO_2_ over CeO_2_-based catalysts. Catal. Today.

[25] Yan B (2016). Dry reforming of ethane and butane with CO_2_ over PtNi/CeO_2_ bimetallic catalysts. ACS Catal..

[26] Theofanidis SA, Galvita VV, Poelman H, Marin GB (2015). Enhanced carbon-resistant dry reforming Fe–Ni catalyst: role of Fe. ACS Catal..

[27] Kim SM (2017). Cooperativity and dynamics increase the performance of NiFe dry reforming catalysts. J. Am. Chem. Soc..

[28] Hohenberg P, Kohn W (1964). Inhomogeneous electron gas. Phys. Rev..

[29] Kohn W, Sham LJ (1965). Self-consistent equations including exchange and correlation effects. Phys. Rev..

[30] Kresse G, Furthmüller J (1996). Efficiency of ab-initio total energy calculations for metals and semiconductors using a plane-wave basis set. Comput. Mater. Sci..

[31] Kresse G, Hafner J (1993). Ab initio molecular dynamics for open-shell transition metals. Phys. Rev. B.

[32] Kresse G, Joubert D (1999). From ultrasoft pseudopotentials to the projector augmented-wave method. Phys. Rev. B.

[33] Blöchl PE (1994). Projector augmented-wave method. Phys. Rev. B.

[34] Perdew JP, Wang Y (1992). Accurate and simple analytic representation of the electron-gas correlation-energy. Phys. Rev. B.

[35] Pack JD, Monkhorst HJ (1976). Special points for Brillouin-zone integrations. Phys. Rev. B.

[36] Henkelman G, Uberuaga BP, Jónsson H (2000). A climbing image nudged elastic band method for finding saddle points and minimum energy paths. J. Chem. Phys..

