# Supplementary material for: Combining CO2 reduction with propane oxidative dehydrogenation over bimetallic catalysts
Source: Nat Commun. 2018 Apr 11;9:1398. doi: 10.1038/s41467-018-03793-w (PMC5893610; doi:10.1038/s41467-018-03793-w)
Supplement: Supplementary file 1 — (DOCX 8938 kb) [file 41467_2018_3793_MOESM1_ESM.docx]

**Supplementary Information**

Combining CO_2_ Reduction with Propane Oxidative Dehydrogenation over Bimetallic Catalysts

Gomez et al.

**Supplementary Methods**

**Catalyst synthesis.** The catalysts evaluated in this study were synthesized through incipient wetness impregnation of metals onto commercially obtained CeO_2_ (35-45 m^2^/g, Sigma-Aldrich). Active metal precursors Pt(NH_3_)_4_(NO_3_)_2_, Ni(NO_3_)_2_∙6H_2_O, and Fe(NO_3_)_3_∙9H_2_O (Alfa Aesar) were dissolved in a volume of water equivalent to the pore volume of an aliquot sample of the support. The metal salt solutions were added by dropwise addition to the support and subsequently mixed. The catalysts were dried at 363 K for 6 hours in order to remove water and to allow the salt to crystallize on the pore surface. Once dried, the catalysts were calcined at 563 K for 2 hours.

The bimetallic catalysts were synthesized by co-impregnation to maximize bimetallic bond formation.^1^ Metal loading amounts utilized 1.7 wt. % Pt as a reference and a 3:1 atomic ratio for Fe:Ni, Fe:Pt, and Ni:Pt. Metal loadings, calcination, and drying treatment conditions are consistent with in-group studies.^2,3^

The average metal particle size of reduced Ni_3_Pt/CeO_2_ was determined to be 2.3 nm from TEM measurements in a previous study.^4^ However, it is very challenging to obtain a reliable particle size distribution for Fe_3_Ni/CeO_2_ using TEM because of the difficulties in measuring 3d elements (Fe and Ni) over the CeO_2_ support. From the in-situ XRD measurements (Supplementary Figure 8), Fe_3_Ni/CeO_2_ did not show any diffraction patterns from metal particles, suggesting that the particle size of Fe_3_Ni should be less than ~2nm.

**Flow reactor calculations.** Flow reactor effluent was analyzed on-stream by a gas chromatograph (GC) equipped with a thermal conductivity (TCD) and flame ionization detector (FID). Argon was used as the inert internal standard to calculate total flow rate and was the basis for all calculations. Blank experiments containing only quartz and treated CeO_2_ were conducted under steady state conditions. These experiments showed little to no activity suggesting that the support, quartz, and gas phase reactions have no significant effect.

In these experiments, conversion is defined as the rate of reactant fed initially into the reactor minus the rate of disappearance of the reactant over the initial rate multiplied by 100:

$Conversion=\frac{{Rate of reactant}_{\mathrm{fed}}-{Rate of reactant}_{\mathrm{consumed}}}{{Rate of reactant}_{\mathrm{fed}}} x 100$

(Supplementary Equation 1)

For CO_2_ and C_3_H_8_, the rate of reactant consumed is defined as:

${Rate of \mathrm{CO}_{2}}_{\mathrm{consumed}}=\frac{Total CO- H_{2}O}{2}+ H_{2}O$

(Supplementary Equation 2)

${Rate of C_{3}H_{8}}_{\mathrm{consumed}}=\frac{Total CO- H_{2}O}{6}+ C_{3}H_{6}$

(Supplementary Equation 3)

In order to compare catalysts according to the available active sites determined by CO chemisorption, the turnover frequency is defined as:

$\mathrm{TOF}_{[\mathrm{site}^{-1}\cdot\min^{-1}]}=\frac{F_{reactant}^{inlet} \times X}{U_{CO} \times W_{catalyst}}$

(Supplementary Equation 4)

where, *F* and *X* are the flow and conversion of a given reactant, respectively, *U_CO_* is the uptake value in mol CO∙g^-1^, and *W* is the mass of catalyst used in g.

For catalysts with low activity, the hydrogen amount was under the detection limit of the GC; therefore, in order to maintain consistency, the selectivity is defined on a C_3_H_8_ basis:

$Selectivity=\frac{Amount of given carbonacious species from C_{3}H_{8}}{Amount of all carbonacious species from C_{3}H_{8}}x100$

(Supplementary Equation 5)

Dehydrogenation activity is based on the amount of propylene produced, reforming is based on the amount of CO produced from 1 mol of C_3_H_8_, and cracking is based on the sum of methane, ethane, and ethylene produced. The CO selectivity is defined as:

(Supplementary Equation 6)

$\mathrm{Selectivity}_{\mathrm{CO}}=\frac{\frac{Total CO- H_{2}O}{2}}{Total Amount of all carbonacious species from C_{3}H_{8}}x100$

Using the selectivity, we can now define the yield on a C_3_H_8_ basis as:

(Supplementary Equation 7)

$\mathrm{Yield}_{\mathrm{product}}=\mathrm{Selectivity}_{\mathrm{product}}\times X_{C_{3}H_{8}}$

**Kinetic measurement methods.** Detailed kinetics experiments for catalysts that exhibit high steady state conversions (≥10%) were performed with approximately 100 mg of diluted (1:5 SiO_2_) and sieved catalyst (40-60 mesh) mixed with roughly 150 mg of treated acid-purified granulated quartz (calcined at 1173 K, 40-60 mesh). Catalysts with low steady state conversion were not diluted. The amount of quartz is adjusted in order to maintain a constant isothermal length in the reactor. Kinetics experiments began with an initial CO_2_ + C_3_H_8_ treatment using the same gas flow ratios and ramp rate as in steady state experiments, but the catalyst bed was held at constant temperature only for 10 hours. Then, the temperature or gas flow was altered depending on the experiment. Conversion values for both reactants were kept well below 10%. Deactivation extents were measured for both apparent activation energy and reaction order experiments by revisiting baseline treatment conditions after obtaining the last experimental point.

Reaction order experiments were conducted after the initial treatment by decreasing and then increasing the flow in increments of 2-2.5 or 5-10 mL/min, depending on the catalyst. The flow was allowed to stabilize for 38 minutes before analyzing the rate. Apparent activation energy experiments were conducted upon the completion of the initial treatment. Then, the reactor temperature was cooled from 823 K to 803 K and then increased by 10 K increments to 833 K. All temperatures were held for approximately 38 minutes. Arrhenius-type plots were constructed by using the rate of dehydrogenation (rate of propylene production) or rate of reforming (CO production from reforming) versus T^-1^. Over Fe_3_Ni, the activation barrier for propane CO_2_ oxidative dehydrogenation was found to be 115 kJ∙mol^-1^, while the activation barrier for reforming over Ni_3_Pt was 119 kJ∙mol^-1^.

**CO chemisorption.** Pulse CO chemisorption was performed to quantitatively compare the number of active sites in each catalyst and for calculating the turnover frequency (TOF). Experiments were performed using an AMI-300ip (Altamira) characterization instrument. Samples were prepared by drying 100 mg of fresh catalyst in a quartz U-tube at 393 K for half an hour with a He flow of 50 mL/min. Once dried, the catalysts were reduced in the presence of 10% H_2_/Ar mixture (30 mL/min total) with a heating rate of 10 K/min from 323 K to 723 K and held for 40 minutes. Reduced samples were then flushed with 50 mL/min of He at 723 K for 20 minutes and allowed to cool to 313 K. Then, pulses of 10% CO/He (590 µL loop) were mixed with the 50 mL/min He stream and passed through the sample. A TCD was used to analyze the amount of gas exiting the bed. The amount of gas adsorbed on the sample is the difference between the amount injected and the amount exiting the bed. Data collection is terminated when the peak area is constant. CO chemisorption results are listed in Table 1 of the main text.

**Thermogravimetric analysis experiments.** TGA experiments were conducted using a TA Instrument 8500 TGA. Approximately 15 mg of spent sample was placed onto a tared weigh pan. The spent catalyst was then subjected to a drying program in which it was ramped from room temperature to 473K at 15 K/min and held at temperature for 45 minutes in the presence of Ar (40 mL/min). Then, the program ramped to 1273 K with a 10 K/min ramp rate in the presence of O_2_ (20 mL/min each). For analysis, the first derivative of the weight loss data (DTGA) is plotted against temperature.

**Transmission electron microscopy (TEM) with energy dispersive spectroscopy (EDS).** EDS can be used to map metal dispersion on catalysts that are difficult to image due to a number of factors such as low metal loading or high support molecular weight. Mapping a sample pre-and post-reaction can help identify whether there are any obvious metal agglomerations that may be caused by sintering. EDS mappings were conducted utilizing an FEI Talos F200X (S/TEM) with an X-FEG field emission source of brightness 1.8x10^9^ A/cm^2^ (at 200 kV). Samples were prepared on gold grids by dropping 20 µL of the supernatant from a 7 mL acetone + 2 mg catalyst mixture that had been sonicated for 20 minutes.

**In-situ X-ray Diffraction (XRD) of Fe_3_Ni/CeO_2_.** In-situ XRD measurements have been added to investigate metal sintering. The sample underwent reduction and reaction treatment at the same temperatures and in proportional gaseous environments as in steady-state experiments. The in-situ data reveal that there are no obvious metal phases, potentially a consequence of low loading. However, it is possible to infer that the metal particles cannot be larger than approximately 2nm, as anything above this would be observed in XRD measurements.

**Density functional theory (DFT) calculations.** Two models were used to describe the Fe_3_Ni cluster supported on CeO_2_. One was the bulk terminated Fe_3_Ni(111) surface using the L12 cubic crystal structure and a four layer 4 × 4 surface slab (Supplementary Figure 10a). The other was the FeO_x_ clusters supported on Ni(111), where both Fe_6_O_9_ and Fe_3_O_3_ clusters on 3 layer 7 × 7 Ni(111) and 5 × 5 Ni(111) surfaces (Supplementary Figures 10c, 12a, and 12b) were considered. In agreement with our experimental observation, the DFT calculations showed that in the presence of oxygen from *CO_2_ dissociation, Fe segregation is thermodynamically more favorable by -0.08 eV/atom than Ni segregation due to the stronger Fe-O bond than Ni-O. Such O-driven Fe segregation can result in the formation of FeO_x_ particles over the surface, resulting in the surface and subsurface layers being Fe-deficient or Ni-rich. Therefore, the selection of FeO/Ni(111) is a reasonable model to represent the FeO/Fe_3_Ni(111) interface. The model of Fe_6_O_9_/Ni(111) is to describe the active interfacial sites. The choice of such small clusters is to achieve a compromise between the computational cost and a reasonable cluster size to explain the trends observed experimentally. The binding energies of intermediates vary with the size of the FeO clusters on Ni(111). However, the overall trend of selectivity may not change. To test this hypothesis, we computed energy profiles for the C-H and C-C bond cleavage using a smaller Fe_3_O_3_/Ni(111) model. It is found that, trends observed using Fe_3_O_3_/Ni(111) are not significantly different from those obtained using Fe_6_O_9_/Ni(111) (Supplementary Figure 12). Since these calculations were computationally demanding, we were unable to test this hypothesis using a FeO cluster larger than Fe_6_O_9_.

Similarly, the Ni_3_Pt cluster supported on CeO_2_ was described by a Ni_3_Pt(111) surface using the L12 cubic crystal structure and a four layer 4 × 4 surface slab (Supplementary Figure 10b), where the formation of the Pt skin on the surface was included to consider the segregation of Pt according to our experimental observations.^4^ In both cases, the effect of CeO_2_ and the particle size was not taken into consideration. According to our experimental results, CeO_2_ plays a crucial role in the initial CO_2_ activation, while the subsequent reforming and dehydrogenation reactions take place on the surface of bimetallic particles. The different trend in the catalytic behavior between Fe_3_Ni/CeO_2_ and Ni_3_Pt/CeO_2_ observed experimentally during propane oxidative dehydrogenation is mostly associated with the surface of bimetallic alloys, which justifies our focus on the trend of different bimetallic surfaces in the DFT calculations.

The Brillouin-zone integration on bulk-terminated Fe_3_Ni(111) and Pt-terminated-Ni_3_Pt(111) and 5 × 5 Ni(111) surfaces was performed on a grid of 3 × 3 × 1 Monkhorst−Pack ^5^ special k-points while the Brillouin-zone integration was sampled at the Γ-point on FeO/Ni(111). A 14 Å thick vacuum was added along the direction perpendicular to the surface in the initial slab model to avoid the artificial interactions between the slab and its periodic images. During geometry optimization, the atoms in the top two layers were allowed to relax while the atoms in the bottom two layers were fixed. The current setup in the DFT calculations predicted the Gibbs free energy (*∆G*) as 1.55 eV for the reaction CH_3_CH_2_CH_3_(g) + CO_2_(g) + *→ CH_3_CHCH_2_(g) + H_2_O(g) + CO(g) + * at T = 298.15 K, which is close to the experimental value of 1.20 eV ^6^.

The binding energy (*BE*) of an adsorbate is calculated as:

*BE(adsorbate) = E(slab + adsorbate) - E(slab) - E(adsorbate),* (Supplementary Equation 8)

where *E(slab + adsorbate)*, *E(slab)* and *E(adsorbate)* are the total energies of the slab with adsorbate, the clean slab, and the adsorbate species in gas phase, respectively.

The transition state of a chemical reaction was located using the climbing image nudged elastic band (CI-NEB) method implemented in VASP.^7^ The activation energy (*E_a_*) of a chemical reaction is defined as the energy difference between the initial and transition states while the reaction energy (*ΔE*) is defined as the energy difference between the initial and final states.

**Supplementary Figures**


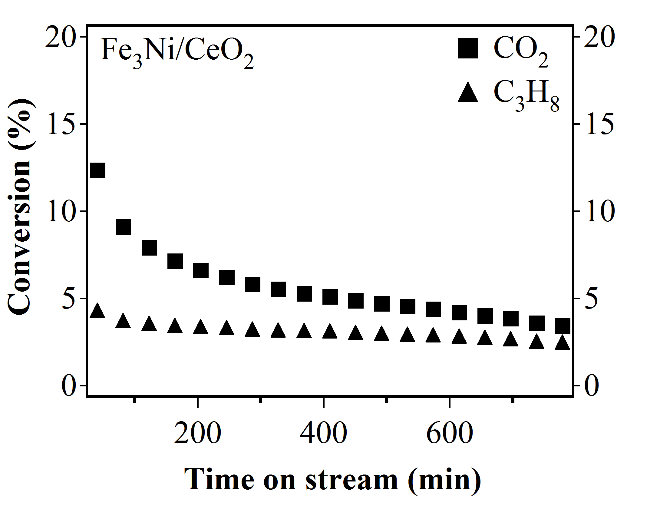


**(a)**


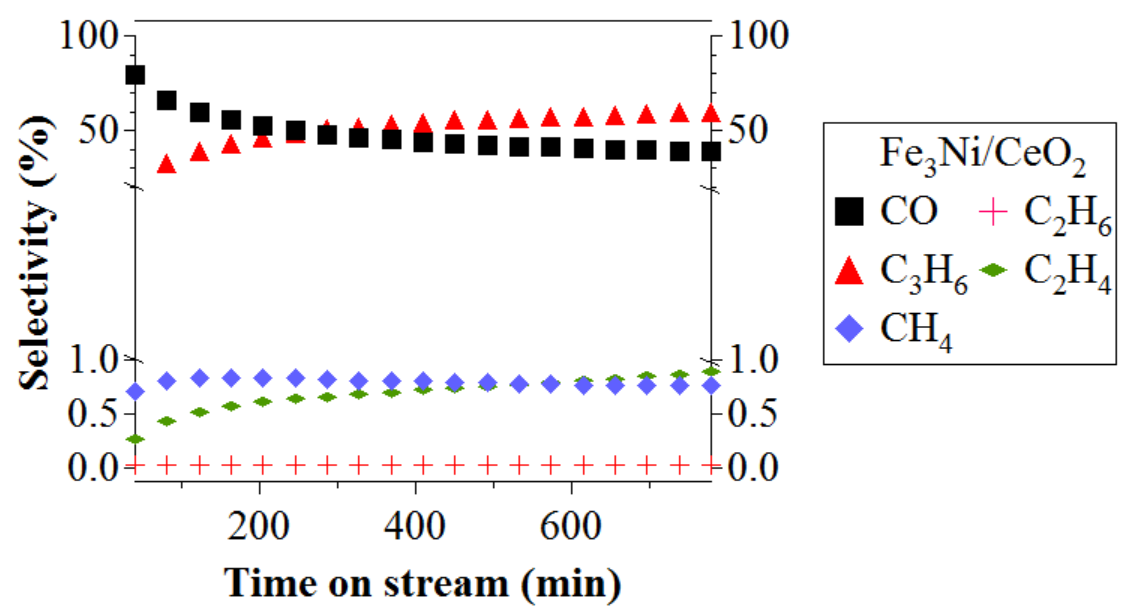


**(b)**


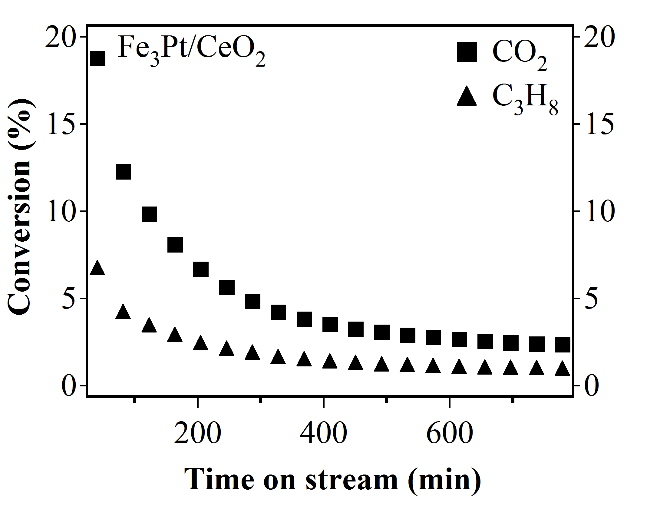


**(c)**


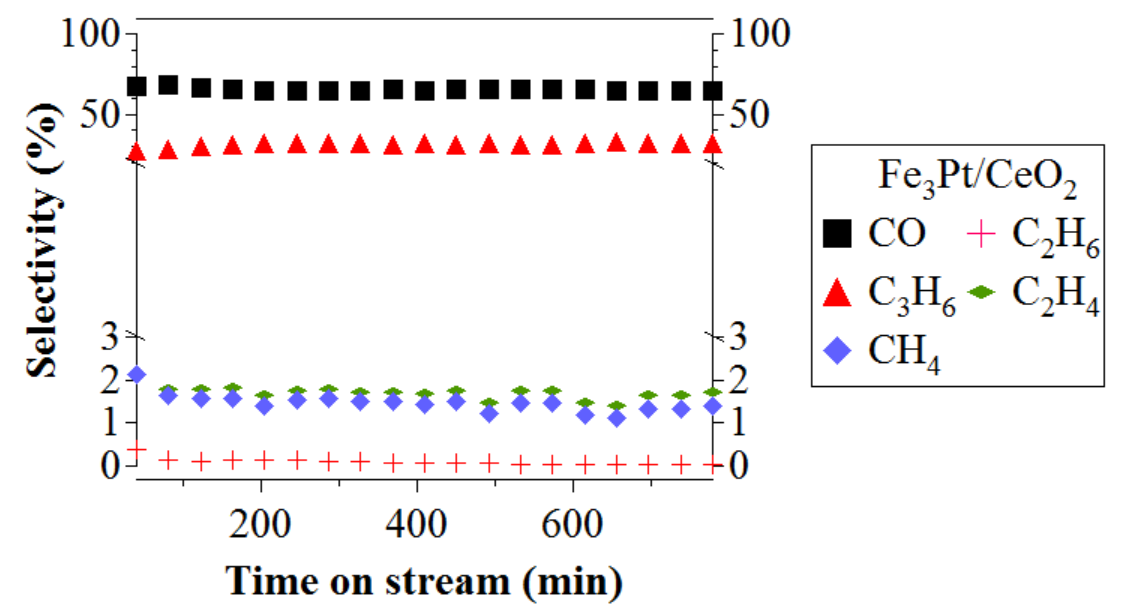


**(d)**


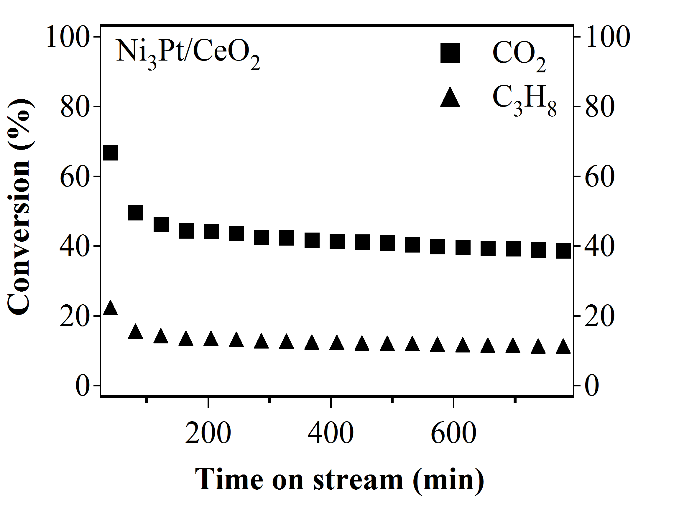


**(e)**


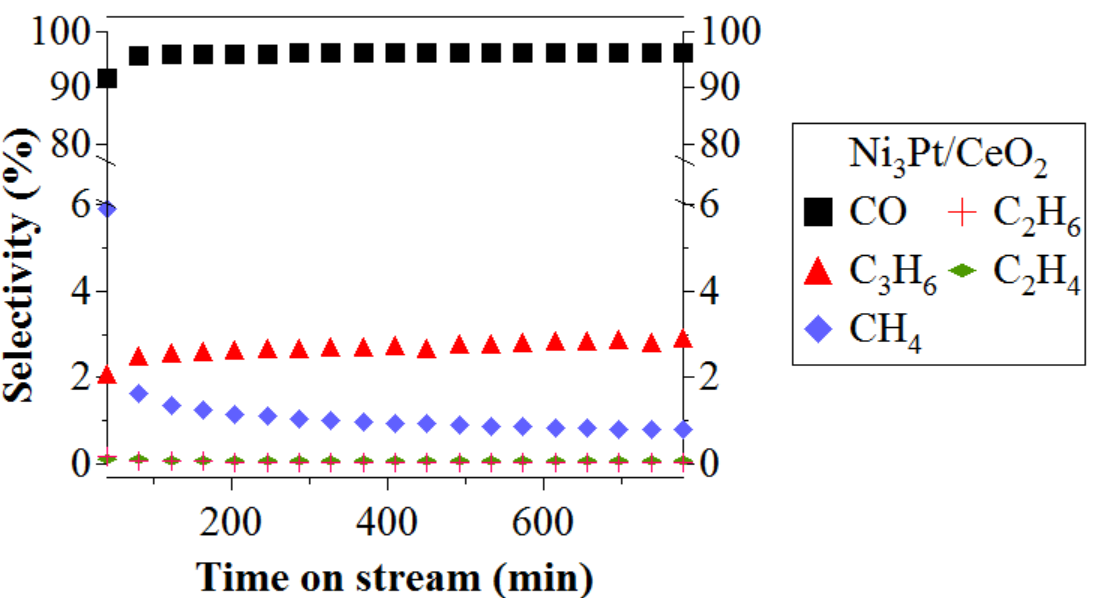


**(f)**

**Supplementary Figure 1***.* Steady-state flow reactor results of reactant conversion and product selectivities following time on stream. (**a,b**) Fe_3_Ni, (**c,d**) Fe_3_Pt, and (**e,f**) Ni_3_Pt for the CO_2_ + C_3_H_8_ reaction (10 mL/min each) at 823 K with Ar diluent (20 mL/min) and 100 mg of catalyst.


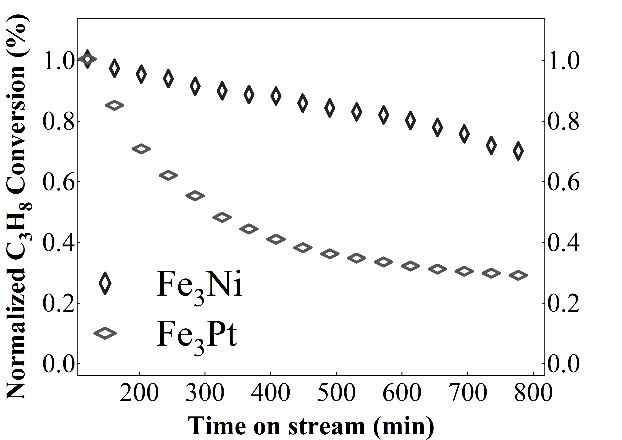

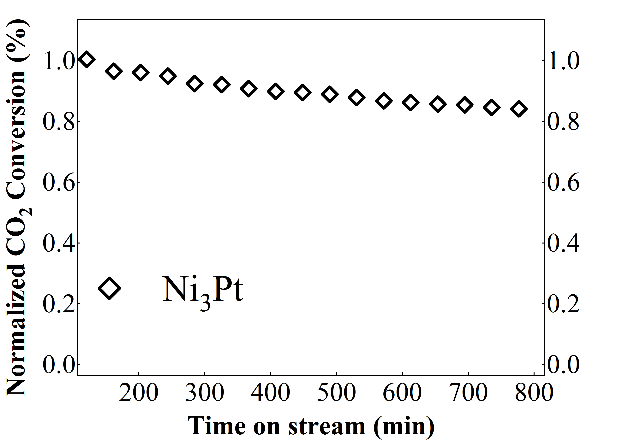


**(b)**

**(a)**

**Supplementary Figure 2.** Normalized reactant conversion following time on stream. (**a**) Fe_3_Ni and Fe_3_Pt (**b**) Ni_3_Pt. Normalization is performed after the initial 123 minutes on stream.


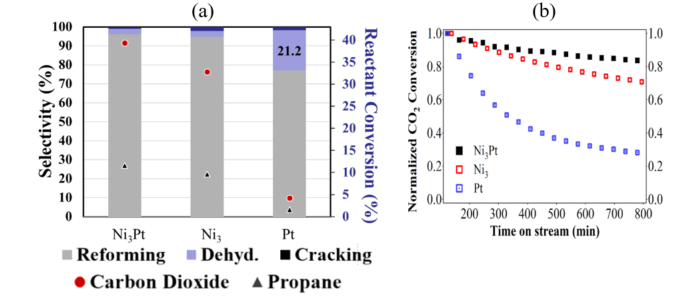


**(a)**

**(b)**

**Supplementary Figure 3**. Comparison among Ni_3_Pt_1_/CeO_2_ and the respective monometallics. Values are for the reaction of CO_2_ + C_3_H_8_ (10 mL/min each) with Ar diluent (20 mL/min) at 823 K and 100 mg of catalyst. (**a**) Selectivity and conversion of Ni_3_Pt, Ni_3_, and Pt_1_ (**b**) normalized CO_2_ conversion vs. time on stream.


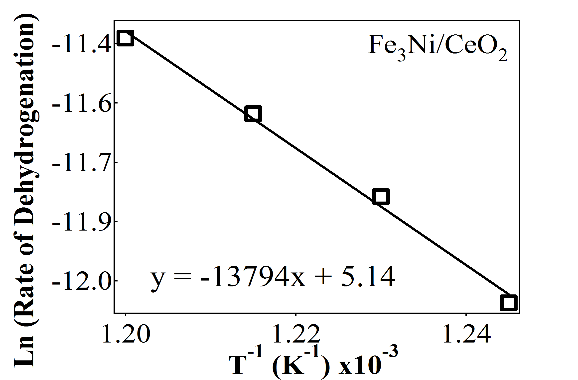


**(a)**


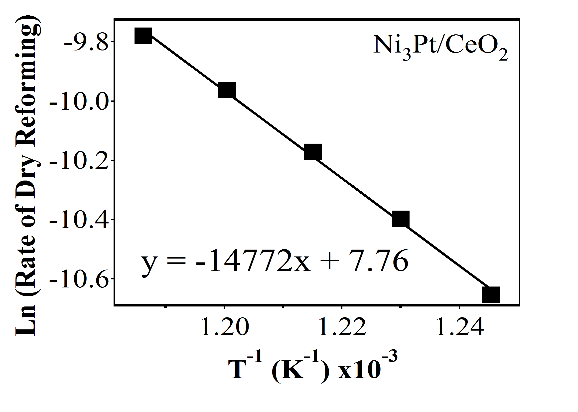


**(b)**

**Supplementary Figure 4.** Apparent activation energy plots. Presented as Ln (rate) versus T^-1^ for (**a**) propane CO_2_ oxidative dehydrogenation over Fe_3_Ni and (**b**) reforming over Ni_3_Pt_._


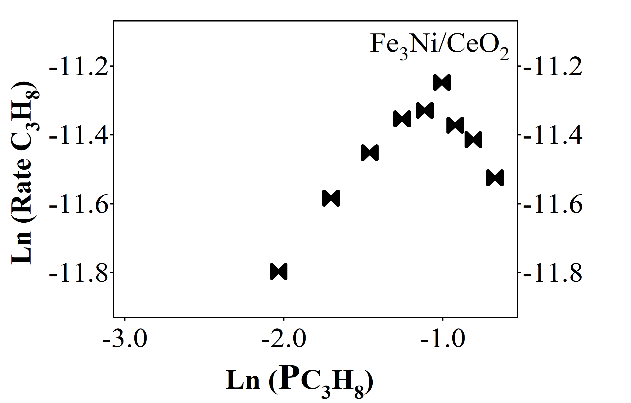

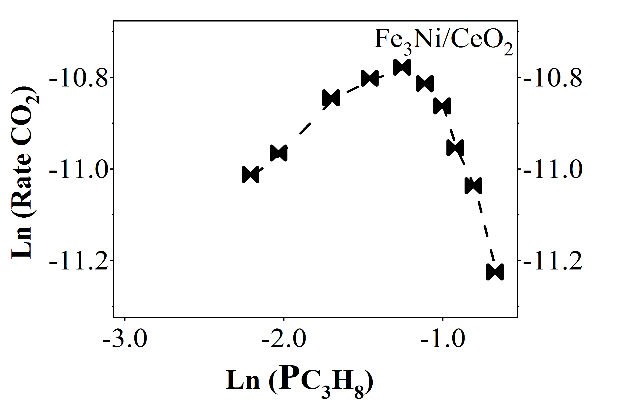

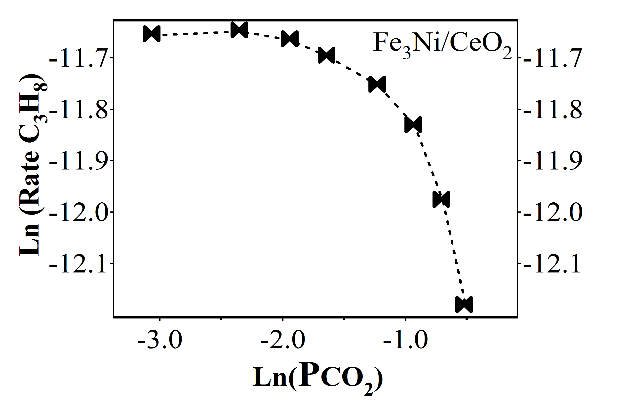

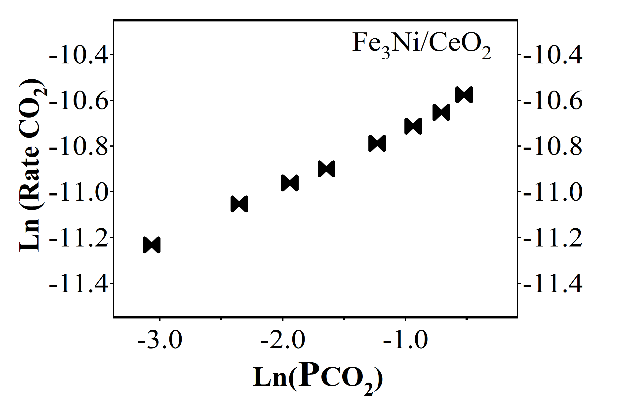


**(a)**

**(b)**

**(c)**

**(d)**


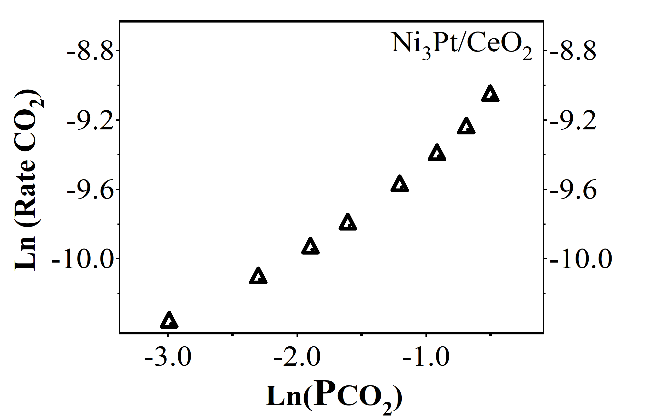

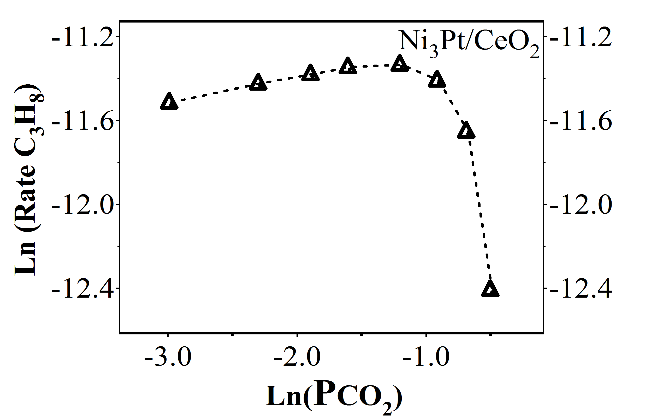

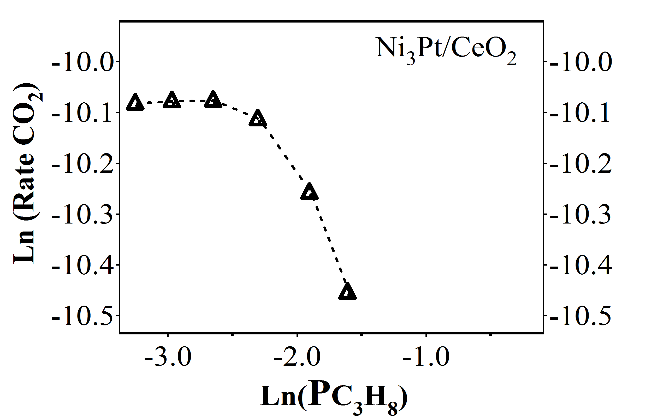

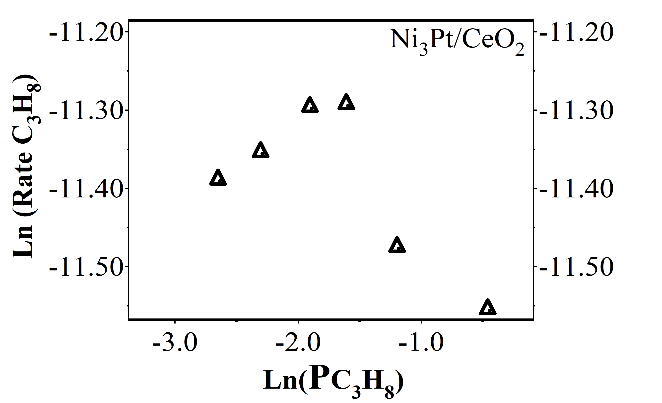


**(e)**

**(f)**

**(g)**

**(h)**

**Supplementary Figure 5.** Effect of reactant partial pressure on reactant consumption rate. CO_2_ partial pressure on the C_3_H_8_ and CO_2_ consumption rate (**a,b**), (**e,f**) and the effect of C_3_H_8_ partial pressure on CO_2_ and C_3_H_8_ consumption rate (**c,d**), (**g,h**) over Fe_3_Ni and Ni_3_Pt, respectively.


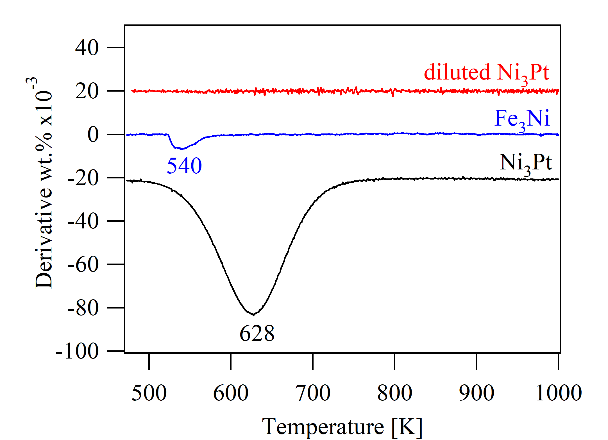


**Supplementary Figure 6.** DTGA results. DTGA for Fe_3_Ni, Ni_3_Pt, and diluted Ni_3_Pt supported on CeO_2_.


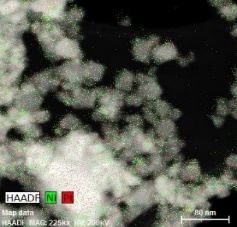

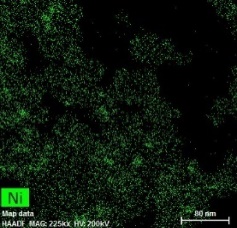

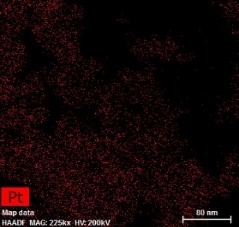

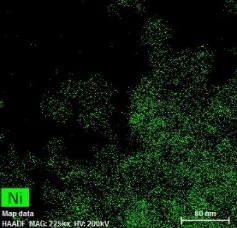

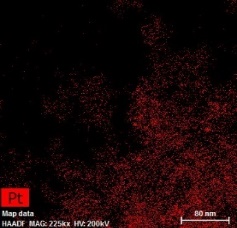

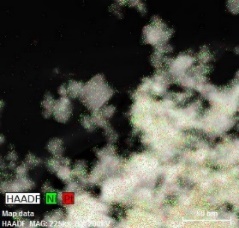


**(b)**


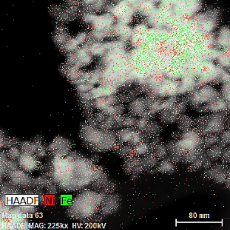

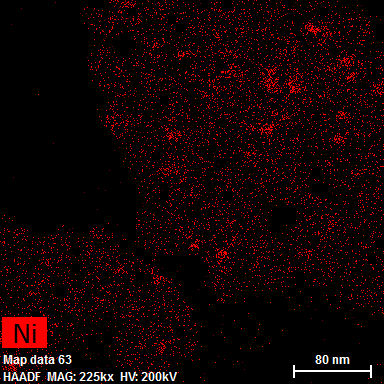

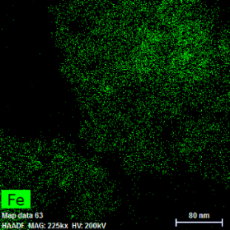

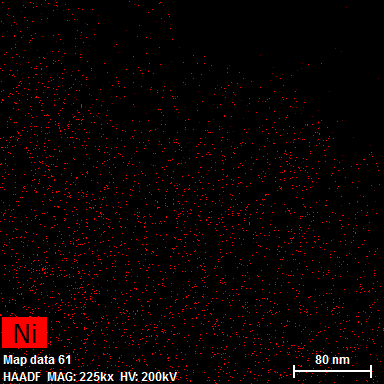

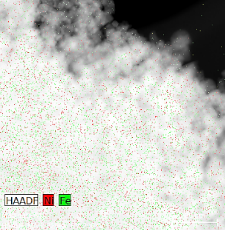

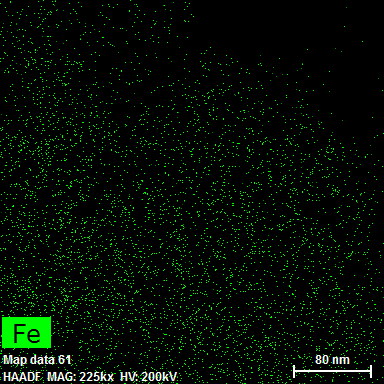


**(a)**

**Supplementary Figure 7.** EDS mapping. (**a**) Fe_3_Ni_1_ and (**b**) Ni_3_Pt. Top sets are reduced catalyst samples while the bottom set are the post reaction samples.


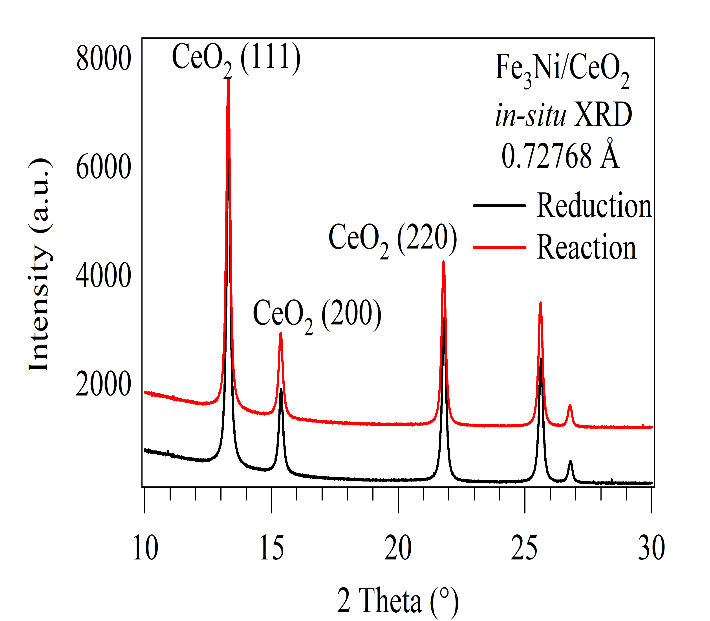

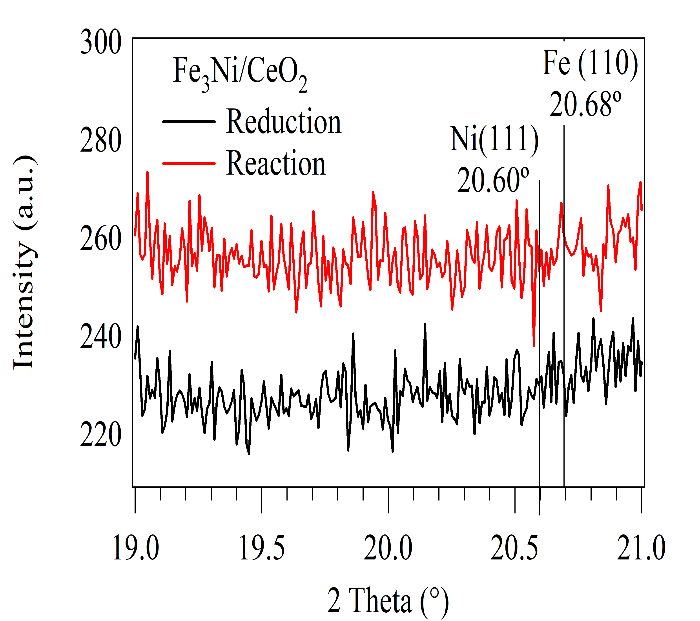

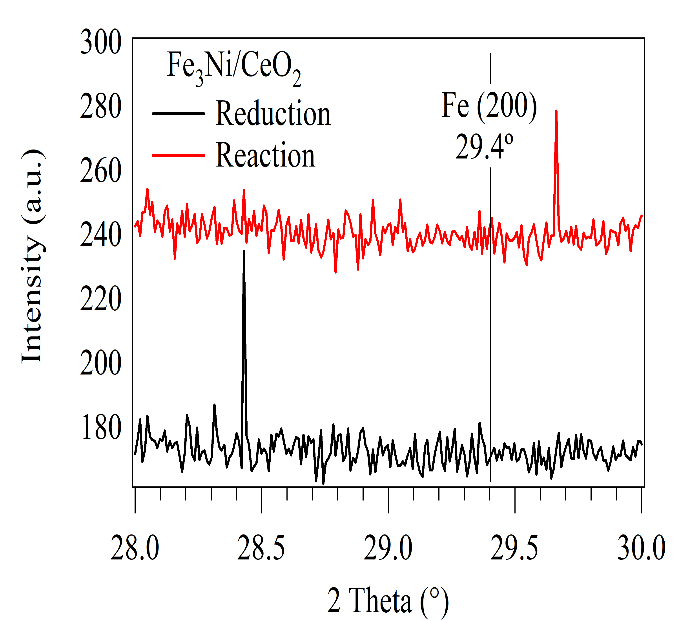

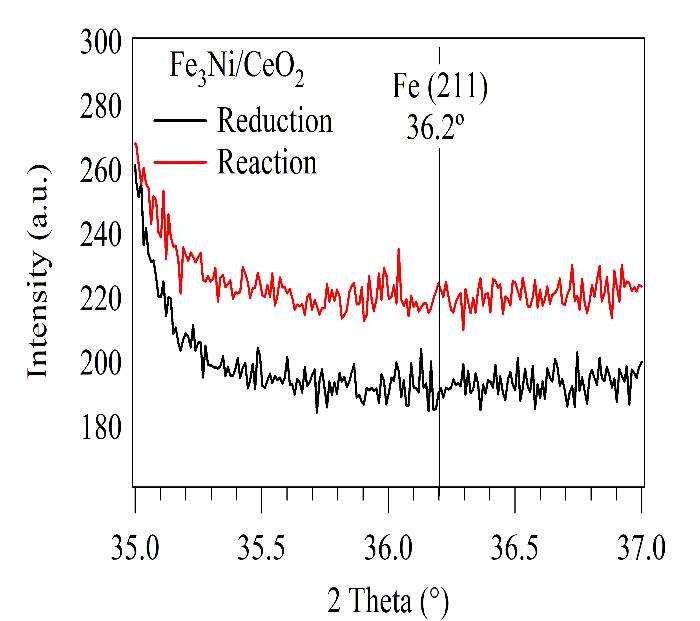


**(a)**

**(b)**

**(c)**

**(d)**

**Supplementary Figure 8.** In-situ XRD intensity vs. 2θ for reduction and reaction treatment. (**a**) Full spectra of Fe_3_Ni/CeO_2_ and (**b-d**) insets for areas of interest with labeled features.


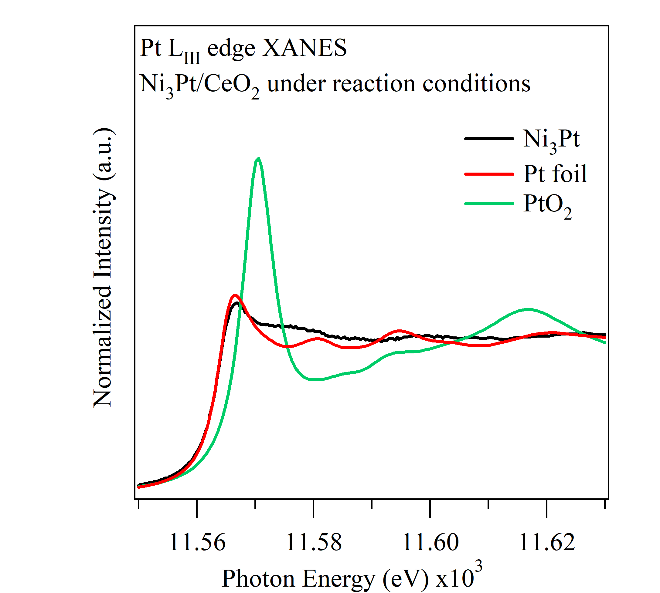


**Supplementary Figure 9.** In-situ XANES spectra. Pt L_III_ edge of Ni_3_Pt/CeO_2_ with respective references.


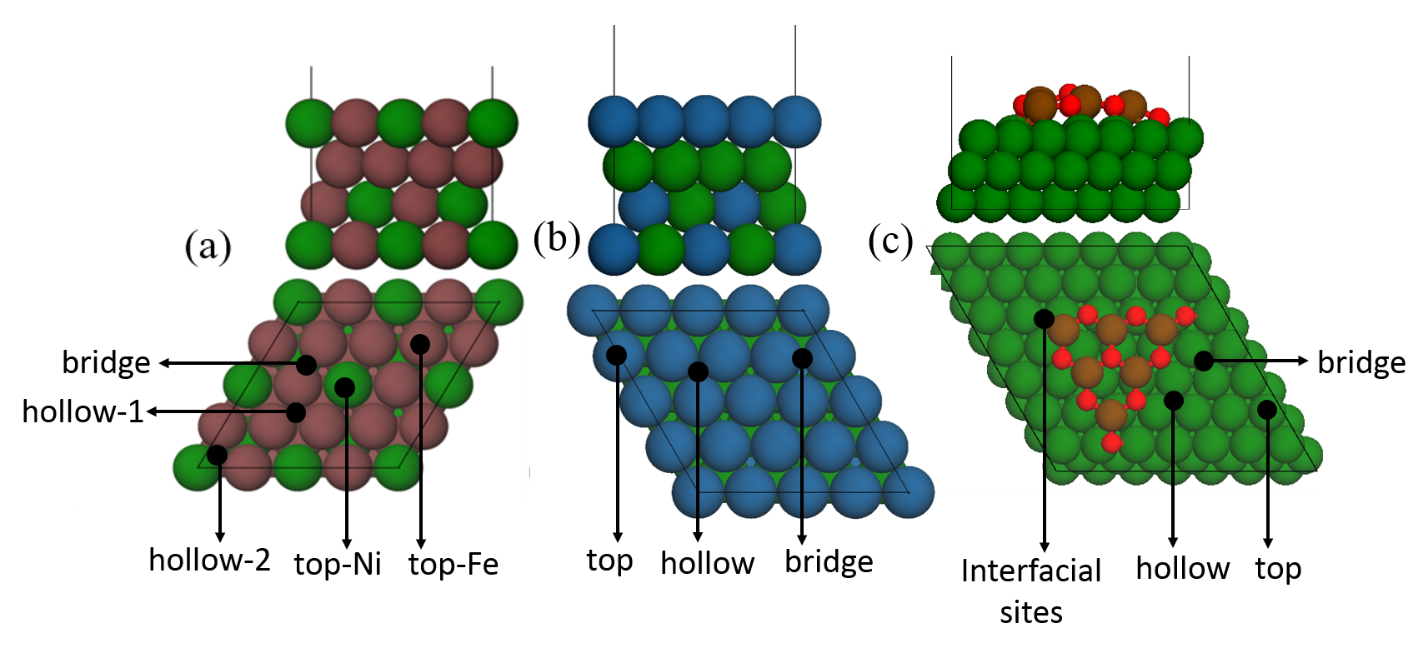


**Supplementary Figure 10.** Optimized geometries of DFT model surfaces. Top image (side) and bottom image (top) views of (**a**) 4 × 4 bulk-terminated Fe_3_Ni(111), (**b**) 4 × 4 Pt-terminated-Ni_3_Pt(111), and (**c**) FeO/Ni(111) unit cells used in DFT calculations. Various adsorption sites are marked by black circle. Pt: blue; Ni: green; Fe: brown; O: red.


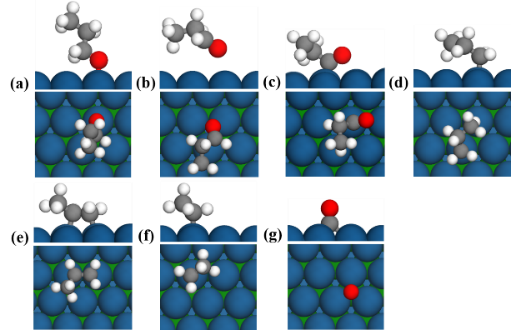


**Supplementary Figure 11.** DFT optimized geometries on the Pt-terminated-Ni_3_Pt(111) surface. Top image (side) and bottom image (top) views of (**a**) CH_3_CH_2_CH_2_O, (**b**) CH_3_CH_2_CHO, (**c**) CH_3_CH_2_CO, (**d**) CH_3_CH_2_CH_2_, (e) CH_3_CHCH_2_, (**f**) CH_3_CH_2,_ and (**g**) CO. Pt: blue, Ni: green, C: grey, O: red, and H: white.


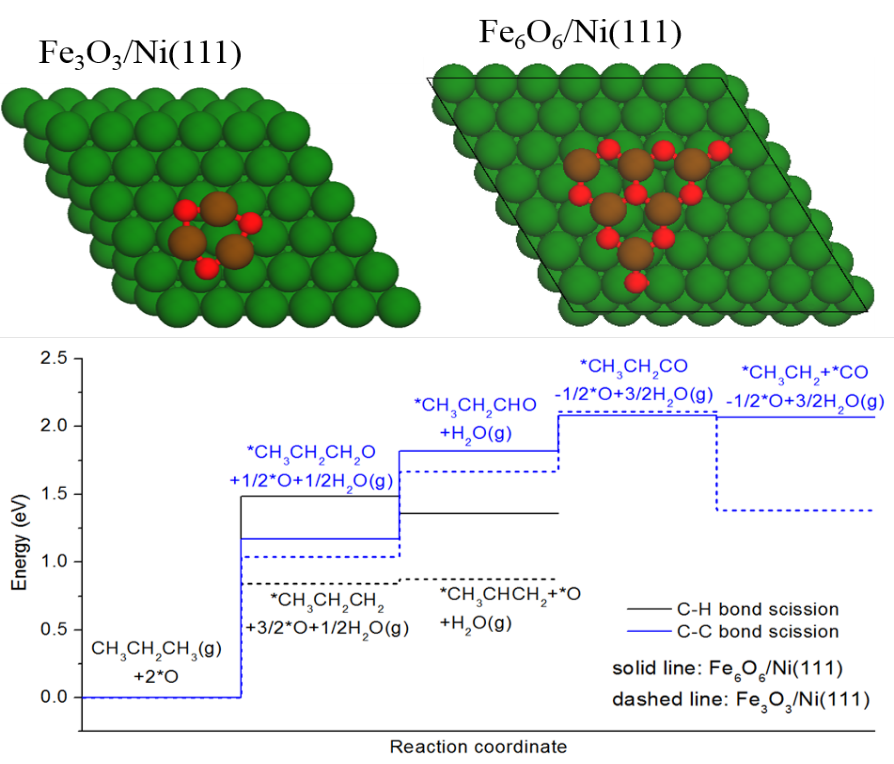


Fe_6_O_9_/Ni(111)

Fe_3_O_3_/Ni(111)

**(a)**

**(b)**

**(c)**

**Supplementary Figure 12.** DFT calculations on the FeO/Ni(111) interface. (**a**) Fe_3_O_3_/Ni(111), (**b**) Fe_6_O_9_/Ni(111), and (**c**) calculated energy profiles for the oxidative C-H and C-C bond scission pathways on both model surfaces.


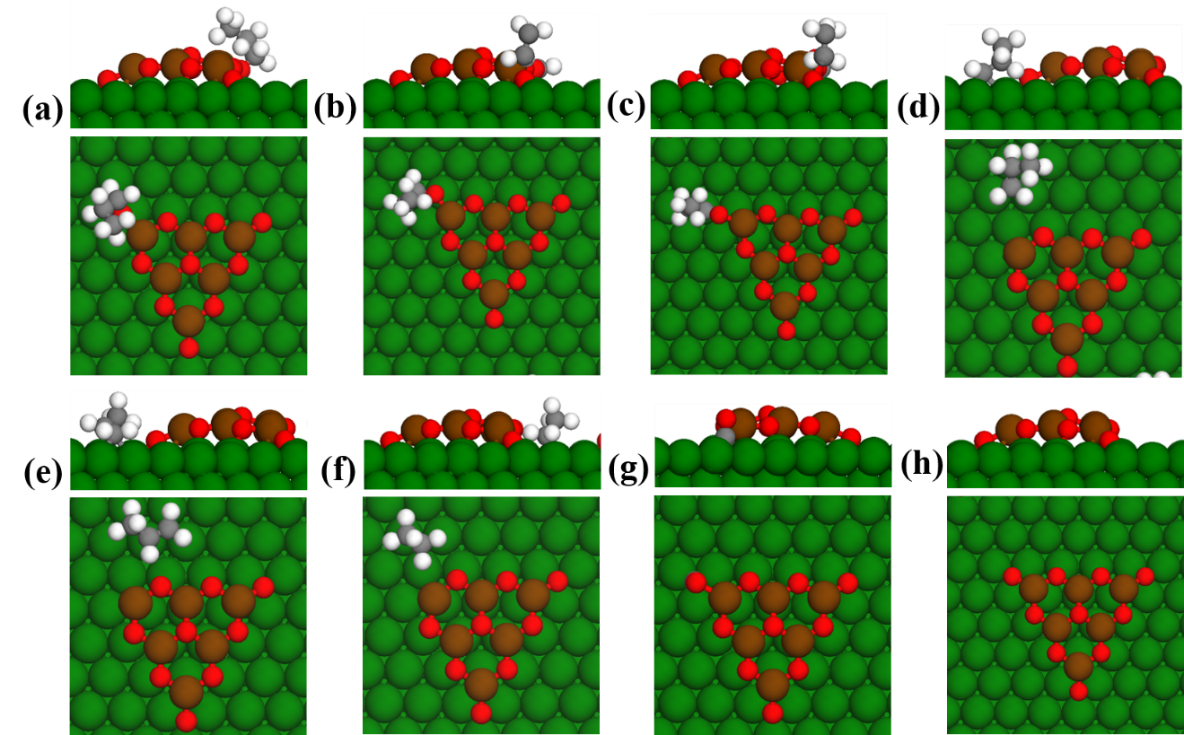


**Supplementary Figure 13.** DFT optimized geometries on FeO/Ni(111). Top (side) and bottom (top) views of (**a**) CH_3_CH_2_CH_2_O, (**b**) CH_3_CH_2_CHO, (**c**) CH_3_CH_2_CO, (**d**) CH_3_CH_2_CH_2_, (**e**) CH_3_CHCH_2_, (**f**) CH_3_CH_2_, (**g**) CO and (**h**) O. Ni: green, Fe: brown, C: grey, O: red, and H: white.


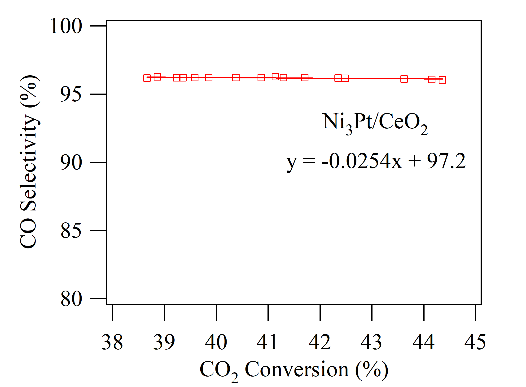

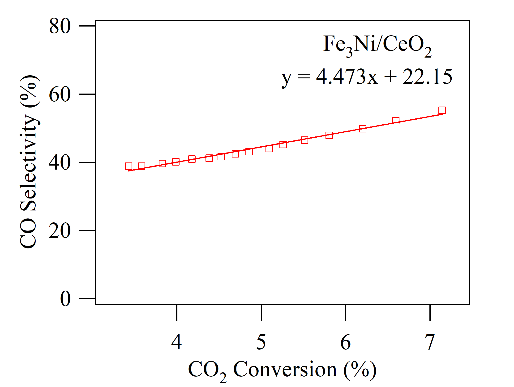


**(a)**

**(b)**


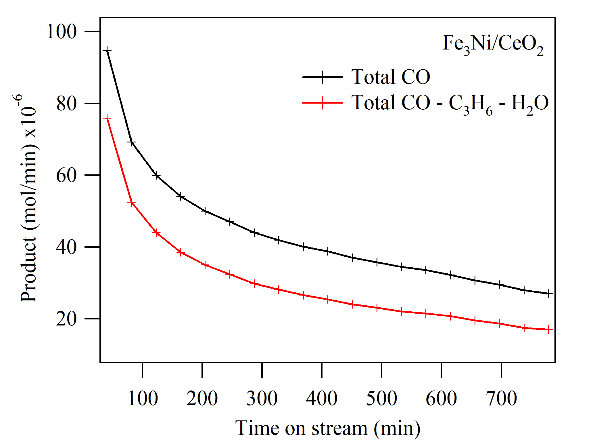
**Supplementary Figure 14**. CO_2_ conversion vs CO selectivity plots. (**a**) Fe_3_Ni and (**b**) Ni_3_Pt bimetallics supported on CeO_2_.

**Supplementary Figure 15*.*** CO production for Fe_3_Ni. Total CO production (top black curve) and total CO production subtracting contributions from CO_2_-ODHP and RWGS (bottom red curve) following time on stream.


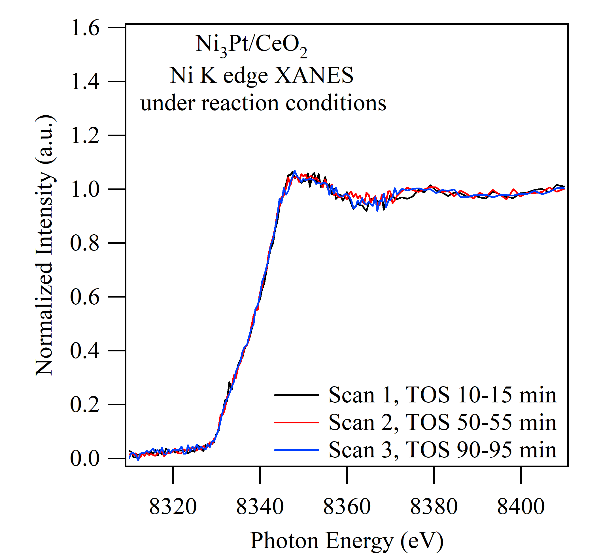

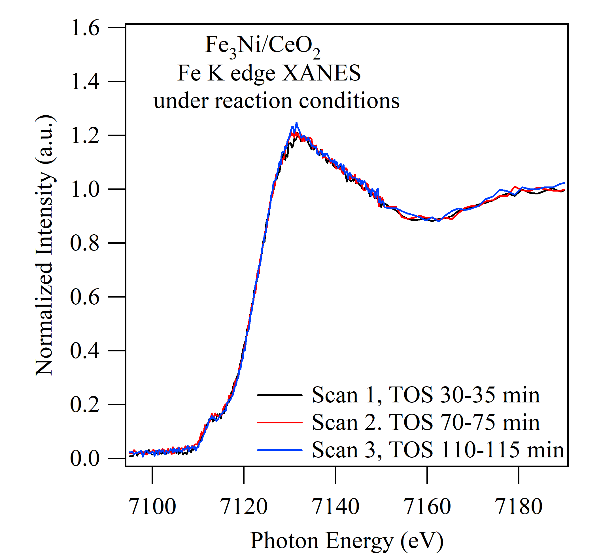

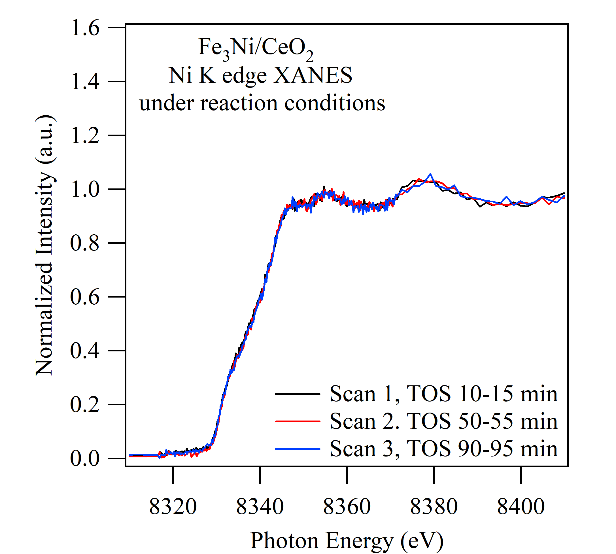

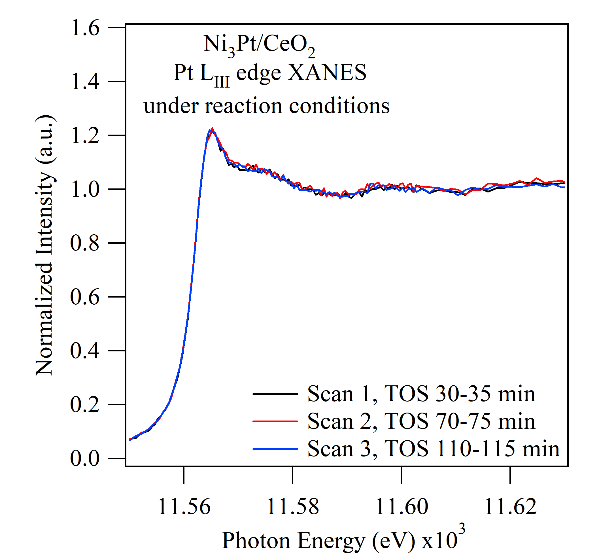


**(a)**

**(b)**

**(c)**

**(d)**

**Supplementary Figure 16.** In-situ reaction scans with indication of time on stream (TOS). Three scans were taken at each edge energy. Ni and Fe K edges for Fe_3_Ni (**a**,**b**) and Ni_3_Pt (**c**,**d**), respectively.


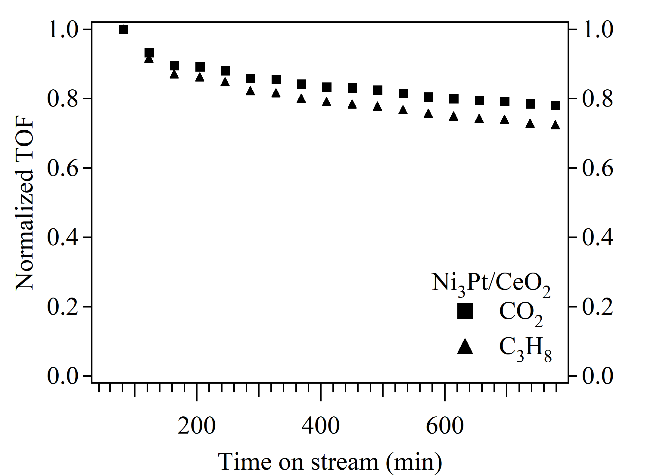


**Supplementary Figure 17.** Normalized TOF. Reactants CO_2_ and C_3_H_8_ over Ni_3_Pt/CeO_2_ following time on stream after 81 minutes. From the three Ni scans in Supplementary Figure 16 it is not evident that there are any changes in oxidation that accompany the observed change in TOF.

**(a)**

**(b)**


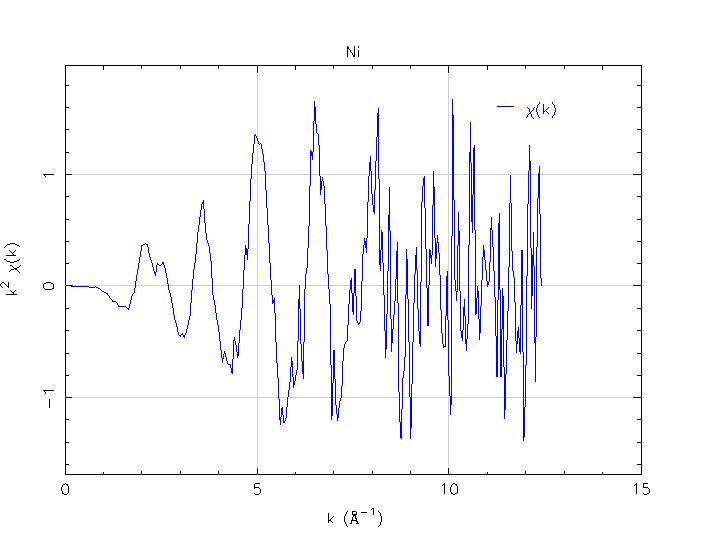

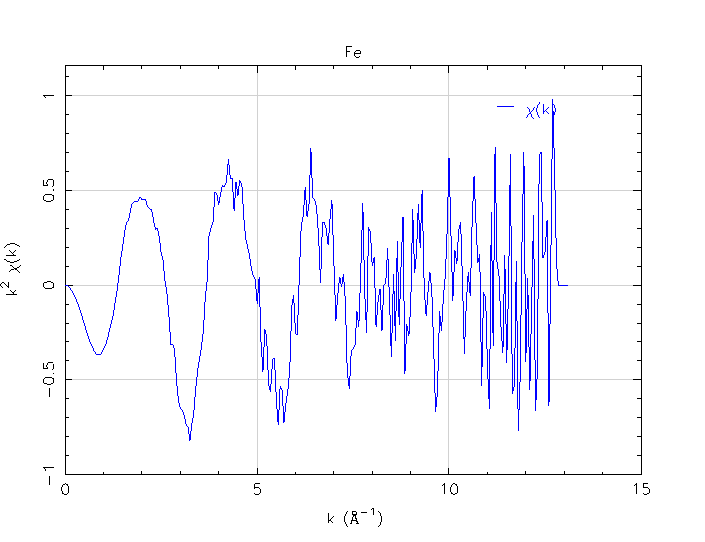


**(c)**

**(d))**

**Supplementary Figure 18***.* EXAFS fitting for Fe_3_Ni/CeO_2._ (**a**) Ni and (**b**) Fe edges. Red hollow circles indicate fitted data. (**c**) and (**d**) k space for the Ni and Fe fitting, respectively.

**Supplementary Tables**

**Supplementary Table 1.** Comparison among Fe_3_Ni_1_/CeO_2_ and the respective monometallics. Values are for the reaction of CO_2_ + C_3_H_8_ (10 mL/min each) with Ar diluent (20 mL/min) at 823 K and 100 mg of catalyst. Steady state conversions of CO_2_ and C_3_H_8_ as well as the C_3_H_6_ and CO yields are included.

| Catalyst | Conversion (%) | | Yield (%) | |
| --- | --- | --- | --- | --- |
| Supported on CeO_2_ | CO_2_ | C_3_H_8_ | C_3_H_6_ | CO |
| Fe_3_Ni_1_ | 4.0 | 2.7 | 1.6 | 1.1 |
| Ni_1_ | 9.3 | 3.0 | 0.4 | 2.6 |
| Fe_3_ | 0.10 | 0.45 | 0.2 | 0.0 |

**Supplementary Table 2.** Comparable C_3_H_8_ reactant conversion with selectivity and yield. Values are for the CO_2_ + C_3_H_8_ reaction (10 mL/min each) at 823 K with Ar (20 mL/min) achieved by diluting (d) the catalyst with treated SiO_2_ in a 1 to 5 ratio_._ Steady state selectivity and yield were on a C_3_H_8_ basis (including only carbonaceous species).

| Catalyst | Conversion (%) | | Selectivity (%) | | | | Yield (%) | | |
| --- | --- | --- | --- | --- | --- | --- | --- | --- | --- |
| Supported on CeO_2_ | CO_2_ | C_3_H_8_ | | CO | C_3_H_6_ | C_1_-C_2_ | | CO | C_3_H_6_ |
| Fe_3_Ni | 4.0 | 2.7 | | 40.2 | 58.2 | 1.6 | | 1.1 | 1.6 |
| Fe_3_Pt | 2.6 | 1.1 | | 65.1 | 32.0 | 2.9 | | 0.7 | 0.3 |
| d-Ni_3_Pt | 7.8 | 2.2 | | 87.8 | 11.0 | 1.2 | | 2.0 | 0.2 |

| Activation Barrier Values (kJ/mol) | | | | |
| --- | --- | --- | --- | --- |
|  | C_3_H_8_ | CO_2_ | C_3_H_6_ | CO |
|  |  |  |  |  |
| Fe_3_Ni /CeO_2_ | 110 | 135 | 115 | 121 |
|  |  |  |  |  |
| Ni_3_Pt/CeO_2_ | 109 | 123 | - | 119 |

**Supplementary Table 3*.*** Activation barrier values for Fe_3_Ni and Ni_3_Pt.

**Supplementary Table 4.** EXAFS summary. EXAFS analysis for the reaction of CO_2_ + C_3_H_8_ over Fe_3_Ni/CeO_2_ and Ni_3_Pt/CeO_2_.

| Sample/Reaction | K Edge | Shell | Bond length (Å) | Coordination Number | σ^2^ (Å^2^) |
| --- | --- | --- | --- | --- | --- |
| Fe_3_Ni/CeO_2_  C_3_ CO_2_-ODH | Ni | Ni-Ni/  Ni-Fe | 2.45+/-0.02 | 10.4+/-0.9 | 0.015 |
|  | Fe | Fe-O | 1.88+/-0.02 | 3.9+/-1.8 | 0.012 |
|  |  | Fe-Fe/  Fe-Ni | 2.88+/0.02 | 1.3+/-0.5 | 0.003 |
|  |  | Fe-O-Fe | 3.53+/-0.03 | 0.7+/-0.4 | 0.003 |
| Ni_3_Pt/CeO_2_  C_3_ CO_2_-ODH | Pt L_III_ | Pt-Ni | 2.56+/-0.02 | 6.4+/-1.4 | 0.010(2) |
|  |  | Pt-Pt | 2.78+/-0.03 | 3.4+/-0.8 | 0.010(2) |

**Supplementary Table 5.** Binding energies. *BE* in eV of adsorbates on various sites shown in Supplementary Figure 10 on bulk-terminated Fe_3_Ni(111).

| Adsorbate | Bound via- | Site | *BE* | Comment |
| --- | --- | --- | --- | --- |
| CH_3_CH_2_CH_2_O | O | top-Fe | -2.63 |  |
|  |  | top-Ni | -- | moved to hollow |
|  |  | hollow-1 | -3.31 |  |
|  |  | hollow-2 | -3.13 |  |
|  |  | bridge | -- | moved to hollow |
| CH_3_CH_2_CHO | C, O | top-Fe_top-Fe | -0.44 |  |
|  |  | top-Fe_top-Ni | -0.74 |  |
|  |  | top-Fe_hollow | -0.86 |  |
|  |  | top-Ni_hollow | -0.78 |  |
|  |  | bridge-bridge | -- | moved to to-hollow |
| CH_3_CH_2_CO | C, O | top-Fe_top-Fe | -2.45 |  |
|  |  | top-Fe_top-Ni | -2.36 |  |
|  |  | top-Fe_hollow | -2.57 |  |
|  |  | bridge-bridge | -2.45 | moved to top-Fe_bridge |
| CH_3_CH_2_CH_2_ | C | top-Fe | -- | moved to hollow |
|  |  | top-Ni | -- | moved to hollow |
|  |  | hollow-1 | -1.80 |  |
|  |  | hollow-2 | -1.74 |  |
|  |  | bridge | -- | moved to hollw |
| CH_3_CHCH_2_ | C, C | top-Fe_top-Fe | -0.67 |  |
|  |  | top-Fe_top-Ni | -- | moved to top-Fe_hollow |
|  |  | top-Fe_hollow | -0.77 |  |
|  |  | bridge-bridge | -0.81 |  |
| CH_3_CH_2_ | C | top-Fe | -- | moved to hollow |
|  |  | top-Ni | -- | moved to hollow |
|  |  | hollow-1 | -2.01 |  |
|  |  | hollow-2 | -1.76 |  |
|  |  | bridge | -- | moved to hollow |
| CO | C | top-Fe | -1.90 |  |
|  |  | top-Ni | -1.62 |  |
|  |  | hollow-1 | -1.80 |  |
|  |  | hollow-2 | -1.92 |  |
|  |  | bridge | -- | moved to hollow |
| O | O | top-Fe | -- | moved to hollow |
|  |  | top-Ni | -- | moved to hollow |
|  |  | hollow-1 | -6.23 |  |
|  |  | hollow-2 | -6.02 |  |
|  |  | bridge | -- | moved to hollow |

**Supplementary Table 6.** Binding energies. *BE* in eV of adsorbates on various sites shown in Supplementary Figure 10 on Pt-terminated-Ni_3_Pt(111).

| Adsorbate | Bound via- | Site | *BE* | Comment |
| --- | --- | --- | --- | --- |
| CH_3_CH_2_CH_2_O | O | top | -1.38 |  |
|  |  | hollow | -- | moved to top |
|  |  | bridge | -- | moved to top |
| CH_3_CH_2_CHO | C, O |  | -0.11 | physisorbed not specific to site |
| CH_3_CH_2_CO | C | top | -2.16 |  |
|  |  | hollow | -- | moved to top |
|  |  | bridge | -- | moved to top |
| CH_3_CH_2_CH_2_ | C | top | -1.70 |  |
|  |  | hollow | -- | moved to top |
|  |  | bridge | -- | moved to top |
| CH_3_CHCH_2_ | C, C | top-top | -0.56 |  |
|  |  | top-hollow | -- | moved to top-top |
|  |  | top-bridge | -- | moved to top-top |
| CH_3_CH_2_ | C | top | -1.82 |  |
|  |  | hollow | -1.75 |  |
|  |  | bridge | -- | moved to top |
| CO | C | top | -1.06 |  |
|  |  | hollow | -1.30 |  |
|  |  | bridge | -- | moved to hollow |
| O | O | top | -- | moved to hollow |
|  |  | hollow | -3.29 |  |
|  |  | bridge | -- | moved to hollow |

**Supplementary Table 7.** DFT calculated reaction energy (*∆E*) and activation energy (*E_a_*) in eV. Shown for selected reaction steps on Pt-ter-Ni_3_Pt(111), bulk-ter-Fe_3_Ni(111) and Fe_3_O_3_/Ni(111).

|  | *CH_3_CH_2_CH_3_ + *O → *CH_3_CH_2_CH_2_ + *OH | | *CH_3_CH_2_CH + *O → *CH_3_CH_2_CH_2_O + * | | *CH_3_CH_2_CH_2_ + *O → *CH_3_CHCH_2_ + *OH | |
| --- | --- | --- | --- | --- | --- | --- |
| Surface | *∆E* | *E_a_* | *∆E* | *E_a_* | *∆E* | *E_a_* |
| Pt-ter-Ni_3_Pt(111) | -0.49 | 1.33 | -0.75 | 1.07 | -0.51 | 1.33 |
| Bulk-ter-Fe_3_Ni(111) | 0.43 | 1.61 | 0.43 | 3.30 | 0.29 | 1.02 |
| Fe_3_O_3_/Ni(111) | 0.63 | 1.88 | 0.01 | 2.13 | -0.40 | 0.29 |

**Supplementary Table 8.** Binding energies. *BE* in eV of adsorbates on various sites shown in Supplementary Figure 10 on Fe_6_O_9_/Ni(111). Since *O and *CO are more strongly bound at the FeO/Ni(111) interface, binding of *C_x_H_y_O species were investigated only at the FeO/Ni(111) interface.

| Adsorbate | Bound via- | Site | *BE* | Comment |
| --- | --- | --- | --- | --- |
| CH_3_CH_2_CH_2_O | O | interface | -2.77 |  |
| CH_3_CH_2_CHO | C, O | interface | -0.88 |  |
| CH_3_CH_2_CO | C, O | interface | -2.47 |  |
| CH_3_CH_2_CH_2_ | C | top | -- | moved to hollow |
|  |  | hollow | -0.96 |  |
|  |  | bridge | -- | moved to hollow |
|  |  | interface | -- | moved to hollow |
| CH_3_CHCH_2_ | C, C | top-top | -- | moved to bridge-bridge |
|  |  | top-hollow | -- | moved to bridge-bridge |
|  |  | bridge-bridge | -0.50 |  |
|  |  | interface | -- | moved to bridge-bridge |
| CH_3_CH_2_ | C | top | -- | moved to hollow |
|  |  | hollow | -1.40 |  |
|  |  | bridge | -- | moved to hollow |
|  |  | interface | -- | moved to hollow |
| CO | C | top | -1.52 |  |
|  |  | hollow | -1.94 |  |
|  |  | interface | -2.04 |  |
| O | O | top |  | moved to hollow |
|  |  | hollow | -5.39 |  |
|  |  | interface | -5.78 |  |

|  | **Fe_3_Ni** | **Fe_3_Pt** | **Ni_3_Pt** | ***Ni_3_Pt** | **Ni_3_Fe** | ***Ni_3_Pt** | **Ni_1_** | **Ni_3_** | **Pt_1_** |
| --- | --- | --- | --- | --- | --- | --- | --- | --- | --- |
| **CO uptake (μmol·g^-1^ )** |  |  |  |  |  |  |  |  |  |
|  | 31.9 | 31.5 | 50.1 | - | 36.0 | - | 13.1 | 37.7 | 16 |
| **Conversion (%)** |  |  |  |  |  |  |  |  |  |
| CO_2_ | 4 | 2.6 | 39.4 | 7.8 | 26.9 | 7.9 | 9.3 | 32.8 | 4.2 |
| C_3_H_8_ | 2.7 | 1.1 | 11.6 | 2.2 | 7.4 | 2.1 | 3 | 9.6 | 1.6 |
| **TOF**  **(site^-1^·min^-1^)** |  |  |  |  |  |  |  |  |  |
| CO_2_ | 5.7 | 3.5 | 37.5 | - | 34.5 | - | 31.9 | 40.2 | 8.1 |
| C_3_H_8_ | 3.4 | 1.5 | 10.5 | - | 9.2 | - | 8.9 | 11.4 | 2.8 |
| **Selectivity (%)** |  |  |  |  |  |  |  |  |  |
| CO | 40.2 | 65.1 | 96.2 | 87.8 | 96.4 | 90.6 | 86.8 | 94.9 | 77 |
| C_3_H_6_ | 58.2 | 32 | 2.8 | 11 | 2.9 | 8.2 | 12.3 | 2.9 | 21.2 |
| CH_4_ | 0.8 | 1.3 | 0.83 | 0.9 | 0.6 | 0.8 | 0.6 | 2.11 | 0.8 |
| C_2_H_6_ | 0 | 0 | 0.1 | 0 | 0.03 | 0 | 0.24 | 0.05 | 0.9 |
| C_2_H_4_ | 0.8 | 1.6 | 0 | 0.3 | 0 | 0.4 | 0 | 0.06 | 0 |
| **Yield**  **(%)** |  |  |  |  |  |  |  |  |  |
| CO | 1.1 | 0.7 | 11.1 | 2 | 7.2 | 1.9 | 2.6 | 9.1 | 1.3 |
| C_3_H_6_ | 1.6 | 0.3 | 0.3 | 0.2 | 0.2 | 0.2 | 0.4 | 0.3 | 0.4 |

**Supplementary Table 9.** Catalyst flow reactor results for CO_2_ + C_3_H_8_ reaction. 10 mL/min each reactant at 823 K with Ar diluent (20 mL/min) and 100 mg of catalyst (16-20 mesh). Catalysts marked with an asterisk indicate that the sample was diluted to achieve comparable C_3_H_8_ reactant conversion to Fe_3_Ni. Values are obtained by averaging data from hours 10-12. Selectivity and yield are on a C_3_H_8_ basis (including only carbonaceous species). Catalysts are synthesized by atomic ratios corresponding to a 1.67 wt.% Pt_1_ basis, thus the weight percent of Fe_3_, Ni_1_, and Ni_3_ are 1.43, 0.5, and 1.5, respectively. The nomenclature assigned by subscripts such as in Fe_3_Ni means that there are 3 atoms of Fe for every atom of Ni.

**Supplementary Notes**

**Supplementary Note 1**

**Comparison of Ni_3_Pt and Fe_3_Ni to Ni_3_Fe.** Furthermore, to compare the activity of Ni_3_Pt with a catalyst containing an equal amount of Ni, Ni_3_Fe was also evaluated. When the atomic ratio of the Fe_3_Ni catalyst is changed to Ni_3_Fe, the catalytic activity becomes higher, leading to 26.9% CO_2_ and 7.4% C_3_H_8_ conversion. However, the reaction pathway favors DRP even at comparable propane conversion (Supplementary Table 9) with a CO selectivity of 90.6%. Thus, when Ni is coupled with non-precious Fe at a ratio of 1:3, higher dehydrogenation selectivity is achieved, and propylene is produced. In contrast, when Ni is alloyed with precious metal Pt, the reforming activity is enhanced compared to Ni_3_Fe and monometallic Ni_3_ (Supplementary Figure 3).

**Supplementary Note 2**

**Relevant reactions and CO_2_ conversion vs. CO selectivity plots.** CO_2_ conversion vs CO selectivity plots for both bimetallics are depicted in Supplementary Figure 14. For Ni_3_Pt, the y-intercept of CO selectivity is 97%, suggesting it is a typical reforming catalyst. While for Fe_3_Ni, the y intercept of CO selectivity is 22.2%. The primary reactions of CO_2_ + C_3_H_8_ include the dry reforming [Supplementary Equation 9] and CO_2_-ODHP [Supplementary Equation 10], which can occur simultaneously at temperatures above 823 K.

| C_3_H_8_ +3CO_2_ → 6CO +4H_2_ | ΔH^o^_R_=622 kJ mol^-1^ | ΔG^o^_R_=384 kJ mol^-1^ | (Supplementary Equation 9) |
| --- | --- | --- | --- |
| C_3_H_8_ +CO_2_→C_3_H_6_+CO+H_2_O | ΔH^o^_R_=166 kJ mol^-1^ | ΔG^o^_R_=119 kJ mol^-1^ | (Supplementary Equation 10) |

Other relevant reactions for propane dry reforming include propane decomposition to carbon, H_2_, and CH_4_ (Supplementary Equation 11), the reverse Boudouard reaction (Supplementary Equation 12), the reverse water-gas shift (Supplementary Equation 13), and direct propane dehydrogenation (Supplementary Equation 14) as listed below.

| C_3_H_8_ → CH_4_ +2C(s) +2H_2_ | ΔH^o^_R_=30 kJ mol^-1^ | ΔG^o^_R_=-26 kJ mol^-1^ | (Supplementary Equation 11) |
| --- | --- | --- | --- |
| CO_2_ + C(s) → 2CO | ΔH^o^_R_=172 kJ mol^-1^ | ΔG^o^_R_=120 kJ mol^-1^ | (Supplementary Equation 12) |
| CO_2_ + H_2_ → H_2_O +CO | ΔH^o^_R_=41 kJ mol^-1^ | ΔG^o^_R_=29 kJ mol^-1^ | (Supplementary Equation 13) |
| C_3_H_8_ → C_3_H_6_ +H_2_ | ΔH^o^_R_=125 kJ mol^-1^ | ΔG^o^_R_=90 kJ mol^-1^ | (Supplementary Equation 14) |

The amount of CO produced from C_3_H_8_ can be calculated via the oxygen balance (i.e., all the oxygen in CO and H_2_O comes from CO_2_),

$F_{CO originated from {CO}_{2}}^{outlet}=\frac{F_{CO}^{outlet}+F_{H_{2}O}^{outlet}}{2}$ (Supplementary Equation 15)

$F_{CO originated from C_{3}H_{8}}^{outlet}=F_{CO}^{outlet}-F_{{CO originated from CO}_{2}}^{outlet}$ (Supplementary Equation 16)

where *F* is the flow rate of reactant or product in mol/min. If no over-oxidation were to occur in the above reaction scheme, then the total CO produced would be equivalent to the sum of C_3_H_6_ and H_2_O produced. However, over the Fe_3_Ni catalyst there is more CO than the sum, (Supplementary Figure 15), indicating that CO can also be produced via over-oxidation of olefins/lighter components and/or the reverse Boudouard reaction. It can be assumed that over-oxidation is included in reforming because all the olefins, lighter components, and C(s) are produced from C_3_H_8_ and are then oxidized by CO_2_ to finally produce CO. Among one of the fundamental differences between CO_2_-ODHP and conventional oxidative dehydrogenation is the net reduction of CO_2_ and the production of CO from over-oxidation, while the latter reaction only produces more CO_2_ upon over-oxidation.

**Supplementary Note 3**

**EXAFS fitting and XANES analysis.** In-situ XAS data collection began after the catalyst had been in contact with reaction gasses and at temperature for 10 minutes. Spectra were collected by alternating between edge energies, starting with the Ni K edge until there were three scans on each edge. The oxidation state of Ni was determined by merging the XANES region of the first (TOS 10-15 min), third (TOS 50-55 min), and fifth (TOS 90-95 min) scans. The XANES scans for Fe K or Pt L_III_ edge were taken at TOS 30-35, 70-75, and 110-115 min. No significant spectroscopic changes were detected on a given edge within the allotted time (Supplementary Figures 16 and 17).

Collecting the fluorescence signal of 3d metals on a support composed by high Z element (CeO_2_ in our system) usually results in relatively weak signals, due to the strong absorption of the oxide support as well as the relatively strong absorption of window materials at low energy edges. For the presented catalytic systems, the Fe and Ce fluorescence signals strongly overlap. The energy range of Ni was also affected by the Cu signal (the in-situ microchannel cell used is made by Cu). In addition, the data were all collected at 873 K and at such high temperature the high thermal displacement would dump the high k data more strongly. Therefore, the data shown in Supplementary Figures 18a and 18b) were noisy above k =10 Å^-1,^ which prevent a good fitting for the relatively weak features between the distance of 2Å-3Å.

In the presented fitting, both Ni and Fe spectra only have about 10 independent points, which could support at most 3-shell fitting (k range from 2.7 to 10.2 with an R range from 1.2 to 3.2~3.6). To reduce the correlation between parameters, constraints and restraints are required. However, since a heterogeneous catalyst is a mixture rather than a compound, there are no good constraints to make. As a result, structural hypotheses are based on the XANES in order not to introduce subjective mistakes. For Ni, metallic Ni foil is used, while for Fe, Fe_2_O_3_ and Fe_3_O_4_ are the models we used.

**Supplementary References**

1. Lonergan, W. W., Wang, T., Vlachos, D. G. & Chen, J. G. Effect of oxide support surface area on hydrogenation activity: Pt/Ni bimetallic catalysts supported on low and high surface area Al_2_O_3_ and ZrO_2_. *Appl. Catal. A Gen.* **408,** 87–95 (2011).

2. Porosoff, M. D. & Chen, J. G. Trends in the catalytic reduction of CO_2_ by hydrogen over supported monometallic and bimetallic catalysts. *J. Catal.* **301,** 30–37 (2013).

3. Myint, M., Yan, B., Wan, J., Zhao, S. & Chen, J. G. Reforming and oxidative dehydrogenation of ethane with CO_2_ as a soft oxidant over bimetallic catalysts. *J. Catal.* **343,**168–177 (2016).

4. Lonergan, W. W., Vlachos, D. G. & Chen, J. G. Correlating extent of Pt–Ni bond formation with low-temperature hydrogenation of benzene and 1,3-butadiene over supported Pt/Ni bimetallic catalysts. *J. Catal.* **271,** 239–250 (2010).

5. Pack, J. D. & Monkhorst, H. J. Special points for Brillouin-zone integrations. *Phys. Rev. B* **13,** 5188–5192 (1976).

6. Dean, J. A. *Lange’s Handbook of Chemistry* (McGraw-Hill, New York,1985).

7. Henkelman, G., Uberuaga, B. P. & Jónsson, H. A climbing image nudged elastic band method for finding saddle points and minimum energy paths. *J. Chem. Phys.* **113,** 9901–9904 (2000).
